# Silver and Copper Dual Single Atoms Boosting Direct Oxidation of Methane to Methanol via Synergistic Catalysis

**DOI:** 10.1002/advs.202302143

**Published:** 2023-07-03

**Authors:** Baiyang Yu, Lu Cheng, Sheng Dai, Yongjun Jiang, Bing Yang, Hong Li, Yi Zhao, Jing Xu, Ying Zhang, Chengsi Pan, Xiao‐Ming Cao, Yongfa Zhu, Yang Lou

**Affiliations:** ^1^ Key Laboratory of Synthetic and Biological Colloids, Ministry of Education, School of Chemical and Material Engineering Jiangnan University Wuxi Jiangsu 214122 China; ^2^ International Joint Research Center for Photoresponsive Molecules and Materials Jiangnan University Wuxi Jiangsu 214122 China; ^3^ Key Laboratory for Advanced Materials and Feringa Nobel Prize Scientist Joint Research Center, School of Chemistry and Molecular Engineering East China University of Science and Technology Shanghai 200237 China; ^4^ Centre for Computational Chemistry and Research Institute of Industrial Catalysis East China University of Science and Technology Shanghai 200237 China; ^5^ Dalian National Laboratory for Clean Energy Dalian Institute of Chemical Physics 457 Zhongshan Road Dalian 116023 China; ^6^ School of Food Science and Technology Jiangnan University Wuxi Jiangsu 214122 China; ^7^ Department of Chemistry Tsinghua University Beijing 100084 China

**Keywords:** dual‐atom centers, methane direct conversion, methanol, selective conversion, synergistic catalysis

## Abstract

Rationally constructing atom‐precise active sites is highly important to promote their catalytic performance but still challenging. Herein, this work designs and constructs ZSM‐5 supported Cu and Ag dual single atoms as a proof‐of‐concept catalyst (Ag_1_−Cu_1_/ZSM‐5 hetero‐SAC (single‐atom catalyst)) to boost direct oxidation of methane (DOM) by H_2_O_2_. The Ag_1_−Cu_1_/ZSM‐5 hetero‐SAC synthesized via a modified co‐adsorption strategy yields a methanol productivity of 20,115 µmol g_cat_^−1^ with 81% selectivity at 70 °C within 30 min, which surpasses most of the state‐of‐the‐art noble metal catalysts. The characterization results prove that the synergistic interaction between silver and copper facilitates the formation of highly reactive surface hydroxyl species to activate the C−H bond as well as the activity, selectivity, and stability of DOM compared with SACs, which is the key to the enhanced catalytic performance. This work believes the atomic‐level design strategy on dual‐single‐atom active sites should pave the way to designing advanced catalysts for methane conversion.

## Graphical abstract

A new kind of Au−Cu dual single atoms supported on ZSM‐5 as an efficient synergistic heterogeneous catalyst for direct oxidation of methane (DOM) to methanol with H_2_O_2_ as oxidant under mild conditions yields a methanol productivity of 20 115 µmol g_cat_^−1^ with 81% selectivity at 70 °C within 30 min.

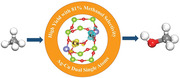

## 1 Introduction

Direct oxidation of methane (DOM) into commercial chemicals and fuels such as methanol and other oxygenates is of paramount importance, and is considered as a potential way to convert methane into high‐value chemicals efficiently and economically.^[^ ^1^, ^2^, ^3^ ^]^ However, the big energy gap between the lowest unoccupied and highest occupied molecular orbitals of CH_4_ molecules makes the cleavage of the first C−H bond of CH_4_ very challenging.^[^ ^4^, ^5^ ^]^ Furthermore, metals such as Pt and Ni with over‐strong adsorption ability of CH_4_ or oxygen species normally induce deep dehydrogenation or over‐oxidation, forming undesired products such as carbon dioxide.^[^ ^6^, ^7^, ^8^ ^]^ Therefore, it is highly desired to simultaneously tune the activation process of CH_4_ and oxygen species for better harvesting value‐added oxygenates.

When the metal particles are downsized to atomic level stabilized by the heteroatom moieties (such as O) of appropriate supports such as oxides and zeolites, the formed M_1_‐O_x_ entity with symmetric charge distribution and isolated site in single‐atom catalysts (SACs) possesses the unique catalytic capability for methane activation and selective conversion.^[^ ^9^, ^10^, ^11^ ^]^ However, the sole adsorption site of SACs generally leads to competitive adsorption and activation when multiple reactants are involved in the catalytic process and the modulation of electronic states of metal centers is normally enslaved to the surface properties of supports, which limits the overall catalytic efficiency of SACs.^[^ ^12^, ^13^ ^]^ Theoretically, the hetero‐SACs by coupling two different single atoms not only enable to modulate the electronic states of metal centers via their electronic interaction but also provide additional adsorption site, thereby improving their catalytic capability in simultaneously modulating the activation process of CH_4_ and oxygen species.^[^ ^14^, ^15^ ^]^

Herein, we report ZSM‐5 supported Cu and Ag dual single atoms (Ag_1_−Cu_1_/ZSM‐5 hetero‐SAC) as a proof‐of‐concept catalyst to demonstrate the unique catalytic behavior of hetero‐SACs for boosting DOM by H_2_O_2_. The switch between mononuclear and dinuclear active site enables to synergistically activate the C−H bond of CH_4_ and the O−O bond of H_2_O_2_ during the process of DOM, which correspondingly yields a methanol productivity of 20115 µmol g_cat_^−1^ and a methanol selectivity of 81% in all products at 70 °C within 30 min as well as good stability (at least five cycles). To our knowledge, the synthesized Ag_1_−Cu_1_/ZSM‐5 hetero‐SAC is one of the most efficient catalysts for DOM to desired methanol compared with that of the state‐of‐the‐art noble and non‐noble metal catalysts under similar reaction conditions as shown in Table S1 (Supporting Information).

## 2 Results and Discussion

### 2.1 Construction and Identification of Ag and Cu Dual Single Atoms in Ag1−Cu1/ZSM‐5 Hetero‐SAC

**Figure 1:**
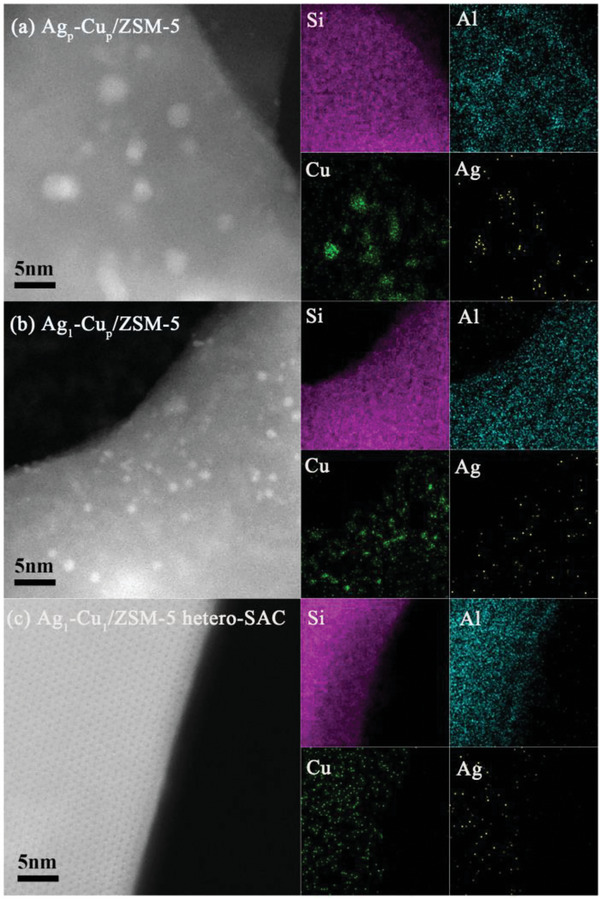
High‐angle annular dark‐field scanning transmission electron microscopy (HAADF‐STEM) images and corresponding elemental maps of a) Ag_p_−Cu_p_/ZSM‐5, b) Ag_1_−Cu_p_/ZSM‐5, and c) Ag_1_−Cu_1_/ZSM‐5 hetero‐SAC (single atom catalyst).

The Ag_1_−Cu_1_/ZSM‐5 hetero‐SAC was synthesized via a modified co‐adsorption method by finely tuning the adsorption parameters of metal precursors in the aqueous solution (details shown in Experimental Section).^[^ ^11^, ^16^, ^17^ ^]^ Aberration‐corrected high‐angle annular dark‐field scanning transmission electron microscopy (HAADF‐STEM) was first utilized to reveal the morphology and structure information of Ag−Cu/ZSM‐5 catalysts. The Ag−Cu nanoparticles can be directly visualized on ZSM‐5 for Ag_p_−Cu_p_/ZSM‐5 from the STEM images (**Figure** **1**a). And the energy‐dispersive X‐ray spectroscopy (EDS) elemental maps further confirm that the Ag and Cu species are both in the form of nanoparticles. The average sizes of metal particles in Ag_p_−Cu_p_/ZSM‐5 are measured to be 3.0 ± 0.8 nm as shown in Figure S1 (Supporting Information). As for Ag_1_−Cu_p_/ZSM‐5 sample, copper species exist as nanoparticles on the surface of ZSM‐5 (Figure 1b). The EDS elemental maps confirm that the Ag species are atomically dispersed on Ag_1_−Cu_p_/ZSM‐5 catalyst (Figure 1b). For the Ag_1_−Cu_1_/ZSM‐5 hetero‐SAC, the corresponding EDS elemental maps show that both silver and copper are atomically dispersed on ZSM‐5 as shown in Figure 1c. More low‐magnification images (Figure S2, Supporting Information) confirm that no nanoparticles or clusters are observed in the Ag_1_−Cu_1_/ZSM‐5 hetero‐SAC, which corroborates the results of EDS. Due to enhanced Z‐contrast, the Ag and Cu atoms can be differentiated under HAADF imaging. Despite some disturbance by the zeolite framework, the intensity profile of the marked sites can still provide a strong indication of adjacent Ag−Cu atomic pairs (average distance of 3.65 Å) by using atomic resolution HAADF‐STEM (Figure S3, Supporting Information). Moreover, the atomically dispersed Cu and Ag species are still stably present and there are no observed nanoparticles or clusters in the used Ag_1_−Cu_1_/ZSM‐5 hetero‐SAC as confirmed by the HAADF‐STEM images (Figure S4, Supporting Information), which reveals the excellent stability of atomically dispersed Ag and Cu species. To conclude, the HAADF‐STEM results clearly show that for the Ag_1_−Cu_1_/ZSM‐5 hetero‐SAC sample, the atomically dispersed silver and copper species are stably anchored on the ZSM‐5.

**Figure 2:**
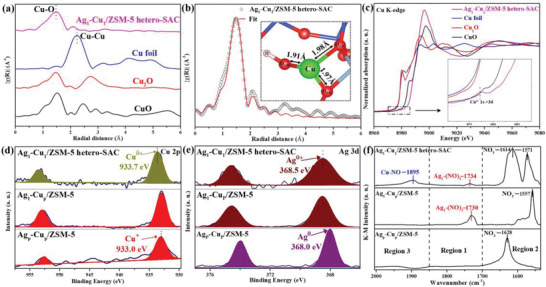
Chemical state and coordination information of Ag−Cu/ZSM‐5 catalysts. *k* ^2^‐weighted µ(*k*) function of extended X‐ray absorption fine structure (EXAFS) spectra (a); EXAFS fitting curve for Ag_1_−Cu_1_/ZSM‐5 hetero‐SAC (single atom catalyst). Inset, proposed coordination environment of Cu_1_‐O_3_ entity (b); Copper K‐edge X‐ray absorption near‐edge structure (XANES) spectra. Inset, pre‐edge of K‐edge region of XANES (c); X‐ray photoelectron spectroscopy (XPS) spectra of Cu 2p for Ag_p_−Cu_p_/ZSM‐5, Ag_1_−Cu_p_/ZSM‐5 and Ag_1_−Cu_1_/ZSM‐5 hetero‐SAC catalysts (d); XPS spectra of Ag 3d for Ag_p_−Cu_p_/ZSM‐5, Ag_1_−Cu_p_/ZSM‐5 and Ag_1_−Cu_1_/ZSM‐5 hetero‐SAC catalysts (e); NO‐DRIFTs (diffuse reflectance infrared Fourier transform spectra) on Ag_p_−Cu_p_/ZSM‐5, Ag_1_−Cu_p_/ZSM‐5, and Ag_1_−Cu_1_/ZSM‐5 hetero‐SAC (f).

Electron paramagnetic resonance (EPR) and UV–Vis spectroscopy were used to further identify the dispersion of copper species. The EPR result (Figure S5, Supporting Information) shows that the g component of Ag_1_−Cu_1_/ZSM‐5 hetero‐SAC is split into a quarter due to hyperfine interactions between unpaired electrons and copper nucleus,^[^ ^18^ ^]^ which indicates that each copper atom is isolated on ZSM‐5 and corroborates the HAADF‐STEM results that copper species are atomically dispersed on Ag_1_−Cu_1_/ZSM‐5 hetero‐SAC. Alternatively, such hyperfine structure is not observed in the Ag_p_−Cu_p_/ZSM‐5, confirming the significant interference between neighboring Cu atoms,^[^ ^9^, ^18^ ^]^ which corroborates the HAADF‐STEM results that copper species exist as nanoparticles in Ag_p_−Cu_p_/ZSM‐5 sample. In addition, the UV–Vis spectra (Figure S6, Supporting Information) reveal that Ag_1_−Cu_1_/ZSM‐5 hetero‐SAC exhibits a wide peak at 204 cm^−1^, which is attributed to the electron transfer of oxygen ligand from ZSM‐5 framework to isolated copper atoms.^[^ ^19^, ^20^, ^21^ ^]^ The X‐ray diffraction pattern of Ag_1_−Cu_1_/ZSM‐5 hetero‐SAC further confirms that silver and copper species are atomically dispersed on ZSM‐5 since there is no observable characteristic peak of copper or silver crystal as shown in Figure S7 (Supporting Information). The in situ NO‐DRIFTs (diffuse reflectance infrared Fourier transform spectra) data also confirms that Ag species in Ag_1_−Cu_1_/ZSM‐5 hetero‐SAC are atomically dispersed, which will be detailedly discussed later in **Figure** **2**. Combining above characterization results, we can clearly conclude that for Ag_1_−Cu_1_/ZSM‐5 hetero‐SAC, silver and copper species are atomically dispersed on ZSM‐5.

### 2.2 Coordination Structure and Chemical State of Ag and Cu Dual Single Atoms in Ag_1_−Cu_1_/ZSM‐5 Hetero‐SAC

Extended X‐ray absorption fine structure (EXAFS) spectroscopy is further performed to characterize the chemical environment of Cu atoms in Ag_1_−Cu_1_/ZSM‐5 hetero‐SAC. Due to the low‐loading (0.0047 wt%) of Ag species in Ag_1_−Cu_1_/ZSM‐5 hetero‐SAC, the data on the local coordination environment of isolated Ag atoms is difficult to directly collect. But for the Cu species, there is only Cu−O scattering centered on 1.46 Å for Ag_1_−Cu_1_/ZSM‐5 hetero‐SAC (Figure 2a), without any Cu−Cu or Cu−O−Cu scattering (from 2.00 to 3.00 Å), which suggests that Ag_1_−Cu_1_/ZSM‐5 hetero‐SAC primarily contains atomically dispersed Cu atoms instead of copper nanoparticles or multicore clusters, and corroborates the conclusion from HAADF‐STEM.^[^ ^9^, ^22^, ^23^ ^]^ To reveal the local coordination environment of Cu species in Ag_1_−Cu_1_/ZSM‐5 hetero‐SAC more clearly, the wavelet transform (WT) of the Cu K‐edge EXAFS oscillations is analyzed. As shown in Figure S8 (Supporting Information), the WT contour plots of Ag_1_−Cu_1_/ZSM‐5 hetero‐SAC present one intensity maximum at approximately 6.0 Å^−1^ that can be assigned to the Cu−O coordination and no intensity maximum related to Cu−Cu or Cu−O−Cu coordination can be observed compared with that of the reference Cu foil, Cu_2_O, and CuO samples. Meanwhile, there is a weak intensity on Ag_1_−Cu_1_/ZSM‐5 hetero‐SAC at approximately 9.8 Å^−1^ that can be assigned to the Cu−Ag scattering and the distance of *Y*‐axis between Ag and Cu atom is about 3.1 Å (phase uncorrected, around 3.6 Å in real space^[^ ^24^ ^]^) in WT, which is in line with the HAADF‐STEM results as shown in Figure S3 (Supporting Information). Those results confirm that the Ag and Cu exist as paired atoms on the Ag_1_−Cu_1_/ZSM‐5 hetero‐SAC and the distance of adjacent Cu Ag_p_ Ag atom pairs is in line with that of the DFT simulated model (3.63 Å).

To accurately reveal the local coordination environment of Cu species in Ag_1_−Cu_1_/ZSM‐5 hetero‐SAC, the standard crystal model built for DFT simulation is used to fit the EXAFS data (Figure S9, Supporting Information). The fitting data fits the original EXAFS data of Cu species well as shown in Figure 2b, which secures that the fitting results are reliable and enable to accurately demonstrate the local structure of isolated Cu atoms in the Ag_1_−Cu_1_/ZSM‐5 hetero‐SAC. The detailed fitting data reveals that Cu atoms are coordinated with one hydroxyl oxygen (O_OH_) and two framework oxygens (O_FM_), respectively. In detail, the bond length of the Cu atom and hydroxyl oxygen (Cu−O_OH_) is 1.91 Å and the average bond length of the Cu atom and framework oxygen (Cu−O_FM_) is 1.98 Å (Table S2, Supporting Information). In addition, when increasing the coordination numbers from 4 to 6 that corresponds to the hydrated Cu species, the significant deviation between the fitting curve and the original data excludes the possibility of Cu atoms existing as hydrated Cu species and further verifies the accuracy of fitted Cu_1_‐O_3_ structure in Ag_1_−Cu_1_/ZSM‐5 hetero‐SAC (Figure S10, Supporting Information).

The electronic states and geometric configurations of Cu species in Ag_1_−Cu_1_/ZSM‐5 hetero‐SAC are further investigated by X‐ray absorption near‐edge structure (XANES). The absorption edge of the Ag_1_−Cu_1_/ZSM‐5 hetero‐SAC sample is higher than that of Cu foil and close to that of CuO as shown in Figure 2c, suggesting that the Cu species carry positive charges and are coordinated with oxygen atoms.^[^ ^25^ ^]^ Besides, there is a pre‐edge peak for Ag_1_−Cu_1_/ZSM‐5 hetero‐SAC (centered at 8977 eV, attributed to the dipole‐forbidden 1 s to 3d electronic transition), which is recognized as the fingerprint of Cu species in a high oxidation state.^[^ ^9^ ^]^ Furthermore, the first derivative of the Cu K‐edge XANES data shows that there is one derivative extremum value found at 8977 eV as shown in Figure S11 (Supporting Information), which further confirms the Cu species in Ag_1_−Cu_1_/ZSM‐5 hetero‐SAC mainly exist as high oxidation state. In addition, the shoulder peak centered at 8986 eV represents the 1s to 4p_z_ transition, which is associated with the typical tetragonal symmetry of Cu species in nanometer‐sized CuO particles.^[^ ^9^, ^26^, ^27^, ^28^ ^]^ Alternatively, the absence of such a characteristic shoulder peak in Ag_1_−Cu_1_/ZSM‐5 hetero‐SAC rules out the existence of the typical geometric configuration of nanometer‐sized CuO particles.^[^ ^29^ ^]^ The EXAFS and XANES results further corroborate the conclusion from HAADF‐STEM that the Ag_1_−Cu_1_/ZSM‐5 hetero‐SAC only contains atomically dispersed Cu species and reveal the high oxidation state of Cu_1_ atoms.

X‐ray photoelectron spectroscopy (XPS) was further used to probe the chemical state of isolated Ag and Cu species (Figure 2d). For Ag_p_−Cu_p_/ZSM‐5, there is one peak centered at 933.0 eV in the Cu 2p_3/2_ signal, which can be assigned to the Cu^+^ species.^[^ ^30^, ^31^ ^]^ For Ag_1_−Cu_p_/ZSM‐5, there is one peak centered at 933.0 eV in the Cu 2p_3/2_ signal, which is the same as that of the Ag_p_−Cu_p_/ZSM‐5 sample and confirms that both Ag_p_−Cu_p_/ZSM‐5 and Ag_1_−Cu_p_/ZSM‐5 samples only contain Cu_2_O nanoparticles.^[^ ^30^ ^]^ For Ag_1_−Cu_1_/ZSM‐5 hetero‐SAC sample, there is one peak centered at 933.7 eV in the Cu 2p_3/2_ signal, higher than that of Cu^+^ species and close to that of Cu^2+^ species, which is correspondingly assigned to the Cu^*δ*+^ (0 < *δ* < 2, close to +2) species and in combination with the XANES results.^[^ ^32^, ^33^, ^34^ ^]^

To further explore the valence state of Cu^*δ*+^, the Mayer bond analysis is further performed for Z[Cu(OH)]^+^[Ag(OH)]^+^ as shown in Figure S12 (Supporting Information). The Mayer bond valences of Cu−O_OH_, Cu−O_FM1_, and Cu−O_FM2_ are 0.92, 0.78, and 0.76, respectively. The formal charge of Cu is also calibrated with reference to the Bader charges of Cu in bulk Cu_2_O (+0.55 |e|) and CuO (+1.08 |e|). The calculated Bader charge of Cu at Z[AgOH]^+^[CuOH]^+^ is +0.98 |e| (Table S3, Supporting Information), which is higher than that of Cu^+^ species and close to that of Cu^2+^ species. Moreover, the Bader charge of hydroxyl (Table S4, Supporting Information) also indicates that OH* can capture an electron from a single Cu atom. And, to maintain the electroneutrality of the whole zeolite, the single Cu atom needs to offer an electron to the framework, resulting in high oxidation‐state copper species. These results confirm that the Cu species in Z[Cu(OH)]^+^[Ag(OH)]^+^ is in a high oxidation state (close to Cu^2+^). These DFT calculation results explain the electronic state of Cu single atoms well, which is in good agreement with the XANES and XPS data.

The XPS data of the Ag_p_−Cu_p_/ZSM‐5 shows that the peak of Ag 3d_5/2_ is centered at 368.0 eV, which can be assigned to the metallic silver (Ag^0^), indicating the nanometer‐sized Ag species are mainly in the metallic state.^[^ ^35^, ^36^ ^]^ Alternatively, the binding energy of Ag species in Ag_1_−Cu_1_/ZSM‐5 hetero‐SAC and Ag_1_−Cu_p_/ZSM‐5 is the same (centered at 368.5 eV) but slightly higher by 0.5 eV compared with that of Ag_p_−Cu_p_/ZSM‐5 (Figure 2e), which can be assigned to Ag^*θ*+^ (0 < *θ* < 1).^[^ ^35^, ^37^, ^38^ ^]^ In short, the chemical states of Ag_1_ atoms and Cu_1_ atoms in Ag_1_−Cu_1_/ZSM‐5 hetero‐SAC suggest that there is a strong electronic interaction between isolated Ag and Cu atoms, which correspondingly modulates the electronic and coordination structure of Cu species as corroborated by EXAFS and XANES data.

To further understand the microscopic structure of the Cu species in the as‐synthesized samples, the experiments of diffuse reflectance infrared fourier transform spectroscopy using NO as a probe molecule (NO‐DRIFTs) were performed on the Ag_p_−Cu_p_/ZSM‐5, Ag_1_−Cu_p_/ZSM‐5, and Ag_1_−Cu_1_/ZSM‐5 hetero‐SAC (Figure 2f).^[^ ^39^, ^40^, ^41^ ^]^ Before each measurement, all the samples are preheated at 423 K for 2 h to remove the possible surface residual adsorbates.

There are two adsorption regions of NO on Ag_1_−Cu_p_/ZSM‐5. The peaks centered at 1730 cm^−1^ in the range of 1850 to 1700 cm^−1^ (Region 1) are associated with NO bound to isolated Ag sites with an adsorption configuration of Ag_1_−(NO)_2_ species,^[^ ^42^ ^]^ which is in line with the HAADF‐STEM results that the Ag species are isolatedly and stably anchored on the ZSM‐5. The peaks centered at 1557 cm^−1^ in the range of 1700 to 1500 cm^−1^ (Region 2) are associated with the NO adsorbed on the surface of ZSM‐5, which is in the form of nitrate ions.^[^ ^43^ ^]^ As for Ag_p_−Cu_p_/ZSM‐5 sample, there is only one peak centered at 1628 cm^−1^ in the range of 1700 to 1500 cm^−1^ (Region 2), which is associated with the adsorption of NO on the surface of ZSM‐5 in the form of nitrate ions.^[^ ^43^ ^]^

For the Ag_1_−Cu_1_/ZSM‐5 hetero‐SAC, there are three adsorption regions of NO as shown in Figure 2f. The peaks centered at 1614 and 1571 cm^−1^ in the range of 1500 to 1700 cm^−1^ (Region 2) are associated with the adsorption of NO on the surface of ZSM‐5 in the form of nitrate ions.^[^ ^43^ ^]^ The peaks centered at 1895 cm^−1^ in the range of 2000 to 1850 cm^−1^ (Region 3) are assigned to NO bound to isolated and high oxidation‐state Cu species with an adsorption configuration of Cu−NO,^[^ ^39^, ^41^ ^]^ which is in line with the XPS and XANES data that the Cu_1_ atoms are mainly in the form of oxidative state. In addition, the peak centered at 1734 cm^−1^ in the range of 1850 to 1700 cm^−1^ (Region 1) is assigned to the asymmetric stretching of NO bound to isolated Ag atom with an adsorption configuration of Ag_1_−(NO)_2_.^[^ ^42^ ^]^ Compared with the Ag_1_−Cu_p_/ZSM‐5 sample, the blue‐shift of Ag_1_−(NO)_2_ on Ag_1_−Cu_1_/ZSM‐5 hetero‐SAC by 4 cm^−1^ (1730 shifted to 1734 cm^−1^) suggests the introduction of Cu single atoms regulates the electronic state structure of Ag species and correspondingly weakens the adsorption strength of NO over Ag sites.^[^ ^42^ ^]^ These results of NO adsorption configuration and position further confirm that Ag species in Ag_1_−Cu_1_/ZSM‐5 hetero‐SAC are atomically dispersed and suggest that the strong electronic interaction between isolated Ag and Cu atoms significantly modulates the electronic state of Ag_1_ and Cu_1_ species, which is in line with the HADDF‐STEM, EXAFS, XPS, and XANES data.

### 2.3 Enhanced Catalytic Activity and Selectivity for DOM via Synergistic Catalysis of Cu and Ag Dual Single Atoms

**Figure 3:**
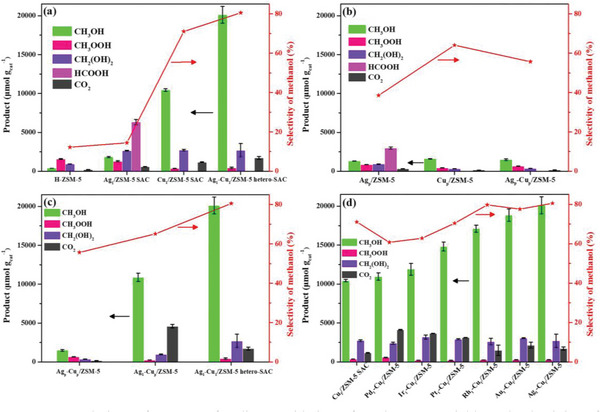
Catalytic performances for direct oxidation of methane (DOM). C1 yields and selectivity of methanol on H‐ZSM‐5, Ag_1_/ZSM‐5 SAC (single atom catalyst), Cu_1_/ZSM‐5 SAC and Ag_1_−Cu_1_/ZSM‐5 hetero‐SAC (a); C1 yields and methanol selectivity on Ag_p_/ZSM‐5, Cu_p_/ZSM‐5 and Ag_p_−Cu_p_/ZSM‐5 (b); C1 yields and methanol selectivity in different dispersion level of Ag−Cu/ZSM‐5 catalysts (c); C1 yields and methanol selectivity of Cu_1_/ZSM‐5 SAC and M_1_‐Cu_1_/ZSM‐5 hetero‐SAC (M represents Pd, Ir, Pt, Rh, Au, and Ag) (d). Reaction Condition: 22 mg catalysts dispersed in 21.05 mL of 0.489 m H_2_O_2_ aqueous solution, 70 °C and 30 bar CH_4_ for 30 min.

To explore the catalytic performance of copper and silver dual single atoms, DOM is performed in a batch reactor by using H_2_O_2_ as the oxidant. The methanol yield of Ag_1_−Cu_1_/ZSM‐5 hetero‐SAC is 20115 µmol g_cat_^−1^ with a methanol selectivity of 81% in all products (the C1 oxygenates yield is 23200 µmol g_cat_^−1^) at 70 °C within 30 min, which surpasses most of the state‐of‐the‐art noble metal catalysts reported in the open literature as shown in Table S1 (Supporting Information). For DOM reaction over pure H‐ZSM‐5 at 70 °C (**Figure**  **3**a), the C1 oxygenates productivity is only 2900 µmol g_cat_^−1^ within 30 min and the major products are the methyl hydroperoxide (55%) and methanediol (32%), around 10 times lower than that of Ag_1_−Cu_1_/ZSM‐5 hetero‐SAC, which indicates that the Ag and Cu species are the main active species. For DOM over ZSM‐5 supported Ag single atoms (Ag_1_/ZSM‐5 SAC) at 70 °C, the yield of C1 oxygenates is 12100 µmol g_cat_^−1^ (Figure 3a), which is much lower than that of Ag_1_−Cu_1_/ZSM‐5 hetero‐SAC. Moreover, the major product from Ag_1_/ZSM‐5 SAC is formic acid (selectivity of 50%) rather than methanol, which indicates that the presence of Cu_1_ atoms as active species determines the conversion direction of DOM over Ag_1_−Cu_1_/ZSM‐5 hetero‐SAC and correspondingly enhances the selectivity of methanol. For DOM over ZSM‐5 supported Cu atoms (Cu_1_/ZSM‐5 SAC) at 70 °C, the yield of C1 oxygenates is 13500 µmol g_cat_^−1^ and the selectivity of methanol is 71% under the same reaction condition, which suggests that the presence of Ag species significantly enhances the catalytic activity for DOM over Ag_1_−Cu_1_/ZSM‐5 hetero‐SAC. Furthermore, the yield of methanol over the physically mixed Ag_1_/ZSM‐5 and Cu_1_/ZSM‐5 (50:50 in wt.%) for DOM is 11700 µmol g_cat_^−1^ with a selectivity of 69%, which is significantly lower than that of Ag_1_−Cu_1_/ZSM‐5 hetero‐SAC (Figure S13, Supporting Information). Those results clearly indicate that there is a significant synergistic effect between neighboring Cu and Ag dual single atoms, which not only boosts the catalytic activity but also promotes the selectivity of methane to methanol.

To further explore such significant synergistic effect for DOM, we probe the catalytic performance of ZSM‐5 supported pure Ag nanoparticle (Ag_p_/ZSM‐5), pure Cu nanoparticle (Cu_p_/ZSM‐5) and Ag−Cu nanoparticle (Ag_p_−Cu_p_/ZSM‐5) catalysts for DOM. The productivity of C1 oxygenates over Ag_p_−Cu_p_/ZSM‐5 (2500 µmol g_cat_^−1^) is similar to that of Cu_p_/ZSM‐5 nanoparticle (2400 µmol g_cat_^−1^) at 70 °C as shown in Figure 3b, which indicates the Ag nanoparticle cannot enhance the catalytic activity of Cu species as what Ag single atoms did on Cu species for DOM. Moreover, the yield of C1 oxygenates over Ag_p_−Cu_p_/ZSM‐5 (2500 µmol g_cat_^−1^) is much lower than that of Ag_p_/ZSM‐5 (6000 µmol g_cat_^−1^) at 70 °C, which suggests that the introduction of copper nanoparticles inhibits the catalytic activity of Ag species for DOM. Those results clearly indicate that there is no significant synergistic effect between Cu and Ag nanoparticles as that of Cu and Ag dual single atoms, which suggests that the synergistic effects between Cu and Ag species are determined by the atomic‐level interaction.

To further verify whether the synergistic effect is determined by the atomic‐level interaction between Cu and Ag species, Ag single atoms with Cu nanoparticle catalyst (Ag_1_−Cu_p_/ZSM‐5) are further prepared for DOM (Figure 3c). The productivity of C1 oxygenates over Ag_1_−Cu_p_/ZSM‐5 (12100 µmol g_cat_^−1^) is around five times higher than that of Ag_p_−Cu_p_/ZSM‐5 nanoparticle catalyst (2500 µmol g_cat_^−1^) at 70 °C, indicating only the atomic‐scale interaction between Ag single atoms and Cu particle can significantly promote the activity of DOM. Moreover, the yield of C1 oxygenates over Ag_1_−Cu_1_/ZSM‐5 hetero‐SAC (23200 µmol g_cat_^−1^) is around two times higher than that of Ag_1_−Cu_p_/ZSM‐5 (12100 µmol g_cat_^−1^) at 70 °C, which further suggests the atomic‐scale interaction between Cu single atoms and Ag species can significantly boost the catalytic activity for DOM.

To better understand the synergistic effect of dual Cu and Ag single atoms, the apparent activation energy (*E*_a_) of the synthesized Ag−Cu/ZSM‐5 catalysts has been investigated. Considering the low conversion rate of methane in DOM, it is difficult to accurately measure the reaction rate of methane and therefore the turnover number (TON) of methanol in fixed reaction time is used as the reaction rate to calculate *E*_a_ for DOM (Figure S14, Supporting Information). A similar method has been reported in previous literature.^[^ ^10^ ^]^ Ag_1_−Cu_1_/ZSM‐5 hetero‐SAC exhibits the lowest *E*_a_ (13.9 kJ mol^−1^) compared with that of Ag_1_−Cu_p_/ZSM‐5 (25.4 kJ mol^−1^) and Ag_p_−Cu_p_/ZSM‐5 nanoparticle (68.9 kJ mol^−1^), which indicates that the catalytic process of DOM is intrinsically boosted on dual Ag and Cu single atoms compared with nanoparticles.

To explore the synergistic effect of hetero‐SACs for boosting DOM, we have further synthesized a series of M_1_‐Cu_1_/ZSM‐5 hetero‐SAC (M represent Pd, Ir, Pt, Rh, Au, and Ag) with similar actual metal loading (see Table S5, Supporting Information) to probe their catalytic performance. As shown in Figure 3d, with the introduction of different noble metal single atoms (Pd, Ir, Pt, Rh, Au, and Ag), the yields of C1 oxygenates gradually increase from 13500 µmol g_cat_^−1^ of Cu_1_/ZSM‐5 SAC to 23200 µmol g_cat_^−1^ of Ag_1_−Cu_1_/ZSM‐5 hetero‐SAC at 70 °C within 30 min, all of which are significantly higher than that of sole Cu_1_/ZSM‐5. In order to understand the changing trend of catalyst activity (as shown in Figure 3d), the methane C−H bonds activation over single atom Cu, dual single atom Pd−Cu and Ag−Cu systems are investigated and compared by DFT calculations (Figure S15, Supporting Information). The free energy barriers of the methane C−H bond activation are sequentially lowered in the order of Cu_1_/ZSM‐5 > Pd_1_‐Cu_1_/ZSM‐5 > Ag_1_−Cu_1_/ZSM‐5, which indicates that the Ag_1_−Cu_1_/ZSM‐5 hetero‐SAC possesses much higher catalytic activity and explains the changing trend observed in Figure 3d. Those results clearly indicate that the hetero‐SACs with dual single atoms as active sites enable to significantly enhance the catalytic performance for DOM compared with sole single atoms. Furthermore, the varied reaction conditions reveal that the reaction temperatures, metal loadings, H_2_O_2_ concentration, and CH_4_ reaction pressures enable to significantly influence the catalytic performance of Ag_1_−Cu_1_/ZSM‐5 hetero‐SAC for DOM (details shown in Figure S16, Supporting Information).

### 2.4 Synergistic Effects of Ag and Cu Dual Sites Toward DOM Reaction Procedures

**Figure 4:**
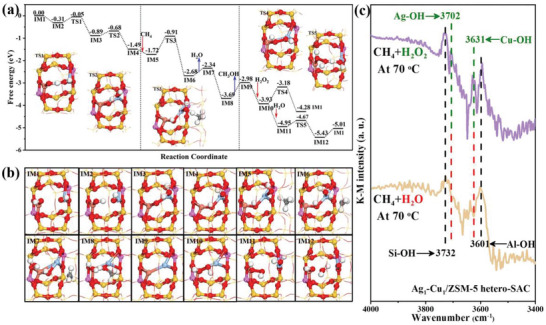
The Gibbs free energy profile of direct oxidation of methane (DOM) over Ag_1_−Cu_1_/ZSM‐5 hetero‐SAC (single atom catalyst) catalyst with the geometry structures of key transition states in three stages of: (i) the transformation from the dual mononuclear site to the dinuclear site, (ii) DOM, and (iii) the regeneration of the dual mononuclear site (a); the geometry structures of all the intermediates in the whole catalytic cycle (b); the in situ FT‐IR spectrum of CH_4_+H_2_O_2_ and CH_4_+H_2_O over Ag_1_−Cu_1_/ZSM‐5 hetero‐SAC at 70 °C (c). The Gibbs energy profile was calculated according to the reaction conditions of 3.0 MPa CH_4_, 0.489 m H_2_O_2_ at 343 K. The H, C, O, Si, Al, Ag, and Cu atoms of the reaction sites are displayed in white, gray, red, yellow, pink, cerulean, and coral in the ball and stick style, respectively.

We further computationally investigate the reaction mechanism of DOM over the Ag_1_−Cu_1_/ZSM‐5 hetero‐SAC at the PBE‐D3 (BJ) level under the reaction conditions as shown in **Figure** **4**a. On the basis of the UV–Vis spectrum, HAADF‐STEM, EXAFS, and WT data of Ag_1_−Cu_1_/ZSM‐5 hetero‐SAC, the active site is a mononuclear structure. The detailed EXAFS fitting data confirms that each Cu atom is coordinated with one hydroxyl oxygen (Cu−O_OH_ bond length of 1.91 Å) atom and two framework oxygen atoms (Cu−O_FM_ bond length of 1.98 and 1.97 Å, respectively), which provides solid evidence to model the active site in Ag_1_−Cu_1_/ZSM‐5 hetero‐SAC (Figure 2b). Meanwhile, the atomic resolution ac‐HAADF‐STEM (Figure S3, Supporting Information) images combined with the WT results (Figure S8a, Supporting Information) provide solid evidence that the average distance between Ag and Cu paired atoms is about 3.65 Å. Based on the above characterization data and catalytic behaviors, several model active sites have been searched (Figure S17, Supporting Information) but only the Z[Cu(OH)]^+^[Ag(OH)]^+^ (IM1 in Figure 4b) is the most reliable reaction site, where the distance of neighboring dual Ag and Cu single atoms is 3.63 Å and each dual single atoms with threefold coordination are located at the *γ*−8MR of ZSM‐5 zeolite without the bonding between Ag and Cu single atoms. In this proposed model active site, the isolated Ag atoms are coordinated with one hydroxyl oxygen atom and two framework oxygen atoms. The detailed bond length and coordination structure of Cu species in the model active site fit the EXAFS data well, which confirms the reliability of the model of the active site used in DFT calculations (Figure 2b and Table S2, Supporting Information). Moreover, the stabilities of different two Al exchange sites to anchor Ag and Cu atoms are systematically calculated. It is found that the required two‐Al exchange site for the formation of the proposed Ag_1_−Cu_1_ site is the most energetically stable one. Hence, these results further verify the reliability of the model of the Ag_1_−Cu_1_ dual‐atom site. The detailed computation is listed in Figure S18 (Supporting Information).

As shown in Figure 4a, the catalytic oxidation of methane using hydrogen peroxide as oxidant at Z[Cu(OH)]^+^[Ag(OH)]^+^ could be divided into three stages: (i) the transformation from the dual mononuclear structure of Z[Cu(OH)]^+^[Ag(OH)]^+^ to the dinuclear structure of Z[Cu(µ‐OH)Ag(OH)]^2+^, (ii) DOM, and (iii) the regeneration of dual mononuclear Z[Cu(OH)]^+^[Ag(OH)]^+^ by H_2_O_2_. The structure transformation starts with the hydrogen atom transfer from [Cu(OH)]^+^ to [Ag(OH)]^+^ via hydrogen bonding. Interestingly, the inverse hydrogen transfer is formidable from [Ag(OH)]^+^ to [Cu(OH)]^+^ due to the unstable [AgO]^+^. Importantly, the hydrogen transfer facilitates the formation of dinuclear Z[Cu(µ‐O)Ag(H_2_O)]^2+^ (IM3). This process only needs to overcome a free energy barrier of 0.26 eV. Although the formed µ‐oxo at IM3 is possible to capture the hydrogen from CH_4_ (*G*_a_ = 0.79 eV) as shown in Figure S19 (Supporting Information), it will preferentially capture the hydrogen from the H_2_O at Ag site (*G*_a_ = 0.21 eV) to generate the active Z[Cu(µ‐OH)Ag(OH)]^2+^ (IM4). Moreover, the desorption of H_2_O from Ag requires an energy of 0.83 eV, which is further in favor of the water oxidative dehydrogenation. Moreover, the whole process of mononuclear‐to‐dinuclear dynamic transformation would release an energy of 1.49 eV, which significantly promotes the stability of the catalyst.

Then, the first C−H bond of CH_4_ is activated by the non‐bridge hydroxyl^[^ ^44^, ^45^, ^46^ ^]^ at the silver ion through the radical‐like mechanism^[^ ^47^, ^48^, ^49^ ^]^ to generate methyl radical and Z[Cu(µ‐OH)Ag(H_2_O)]^2+^ (IM6), which needs to overcome a free energy barrier of 0.81 eV (TS3). The water molecule at Z[Cu(µ‐OH)Ag(H_2_O)]^2+^ can easily desorb from silver ions. Then, the generated methyl radicals can be readily captured by the bridged hydroxyl at Z[Cu(µ‐OH)Ag]^2+^, which is energetically lowered by 1.01 eV. The dinuclear Z[CuAg]^2+^ (IM9) is then generated after the desorption of methanol.

Moreover, the Z[CuAg]^2+^ (IM9) enables the facile O−O bond activation of H_2_O_2_ to form the Z[Cu−HO−OH−Ag]^2+^ (IM10) by releasing an energy of 0.95 eV, which is a significantly exothermic process. And then with the assistance of H_2_O, the dual mononuclear of Z[Cu(OH)]^+^[Ag(OH)]^+^ active site can be easily regenerated by overcoming a rather facile free energy barrier of 0.28 eV (barrier of IM11 to TS5). Hence, it is clear that the methane conversion catalyzed by Ag_1_−Cu_1_/ZSM‐5 hetero‐SAC depends on the cooperation of Ag_1_ and Cu_1_ sites to activate the C−H bond of methane and the O−O bond of H_2_O_2_ during the process of DOM. It should be noted that H_2_O_2_ is the key to regenerate the active size and H_2_O plays an assisting role for H_2_O_2_ in the whole regeneration process, which is further proved by the activity experiment. Under the experimental conditions without H_2_O_2_ species, only trace methanol can be detected in the liquid phase (sole H_2_O as reaction medium) over Ag_1_−Cu_1_/ZSM‐5 hetero‐SAC after DOM reaction (Figure S20, Supporting Information), which indicates that the presence of H_2_O_2_ is the key to the regeneration of Z[Cu(µ‐OH)Ag(OH)]^2+^ (IM4) active site.

In order to further detect the formation process of hydroxyl species on isolated Ag and Cu atoms, the in situ DRIFTS of reactive gas (CH_4_+H_2_O_2_ and CH_4_+H_2_O) is conducted as shown in Figure 4c. When the H_2_O_2_ solution (0.489 m) is introduced by methane into the reaction system, four vibration peaks of the O−H bond can be observed on the FT‐IR spectrum. The peaks centered at 3732 and 3601 cm^−1^ are associated with OH group adsorbed on isolated Si and Al sites, respectively ;^[^ ^40^, ^50^, ^51^, ^52^ ^]^ the peaks centered at 3702 and 3631 cm^−1^ are associated with OH group adsorbed on Ag (Ag−OH) and Cu (Cu−OH) sites, respectively,^[^ ^23^, ^40^, ^41^, ^53^ ^]^ which clearly suggests that the H_2_O_2_ can be easily dissociated to hydroxyl species on Ag and Cu sites. Alternatively, only when H_2_O is introduced into the reaction system, the above Ag−OH and Cu−OH groups are absent. Those results suggest that H_2_O_2_ species plays a key role in the process of generating the hydroxyl species and regenerating the dual active sites (Z[Cu(OH)]^+^[Ag(OH)]^+^), which corroborates the DFT calculation results (process iii).

**Figure 5:**
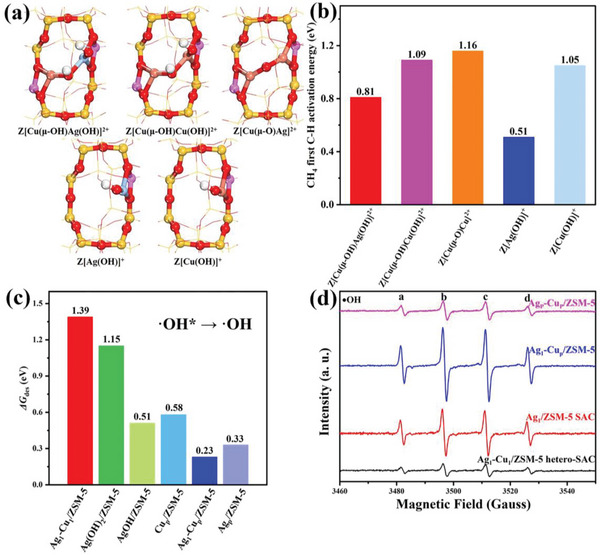
The geometry structures of the Z[Cu(µ‐OH)Ag(OH)]^2+^, Z[Cu(µ‐OH)Cu(OH)]^2+^, Z[Cu(µ‐O)Cu]^2+^, Z[Ag(OH)]^+^, and Z[Cu(OH)]^+^ sites (a); the first C−H bond activation free energy of methane on each structure (b); the desorption free energy of hydroxyl radical on active sites (c); electron paramagnetic resonance (EPR) spectra of hydroxyl radicals trapped by 5, 5’‐dimethyl‐1‐pyrroline *N*‐oxide (DMPO) of Ag_1_−Cu_1_/ZSM‐5 hetero‐SAC (single atom catalyst), Ag_1_/ZSM‐5 SAC, Ag_1_−Cu_p_/ZSM‐5, and Ag_p_−Cu_p_/ZSM‐5 (d).

To further understand the catalytic role of Cu and Ag dual single atoms in boosting the catalytic performance for DOM and to explore the possible binulcear Cu‐oxo species formed through the Cu ions migration,^[^ ^54^ ^]^ we further computationally compare the catalytic processes of DOM over Z[Cu(µ‐OH)Ag(OH)]^2+^, Z[Cu(µ‐OH)Cu(OH)]^2+^, Z[Cu(µ‐O)Cu]^2+^, Z[Ag(OH)]^+^, and Z[Cu(OH)]^+^ sites (**Figure** **5**a). Since the first C−H bond activation of CH_4_ is the rate‐determining step for DOM over Ag_1_−Cu_1_/ZSM‐5 hetero‐SAC, the free energy barrier is used as the descriptor to evaluate the activities of these sites. As depicted in Figure 5b, the order of the free energy barriers for the C−H bond activation follows Z[Ag(OH)]^+^ < Z[Cu(µ‐OH)Ag(OH)]^2+^ < Z[Cu(OH)]^+^ < Z[Cu(µ‐OH)Cu(OH)]^2+^ < Z[Cu(µ‐O)Cu]^2+^. Notably, the existence of the Ag_1_ atom and the synergistic effects between Cu and Ag dual single atoms effectively facilitate the C−H bond activation. Furthermore, the difference in the activation free energies of the C−H bond of CH_4_ and CH_3_OH ($G_{a}^{{\mathrm{CH}}_{4}}$ and $G_{a}^{{\mathrm{CH}}_{3}\mathrm{OH}}$) can be used to demonstrate the selectivity of methane to methanol.^[^ ^55^ ^]^ The minimum C−H bond activation free energy gap between CH_4_ and CH_3_OH at Z[Cu(µ‐OH)Ag(OH)]^2+^ (Figure S21, Supporting Information) indicates that the synergistic effects between Cu and Ag dual single atoms could also improve the selectivity toward the formation of methanol compared with Ag_1_/ZSM‐5 SAC. This is in good agreement with our experimental results that the Ag and Cu dual atoms in Ag_1_−Cu_1_/ZSM‐5 hetero‐SAC enable to significantly enhance the catalytic performance for DOM compared with the sole Ag and Cu as the active site in Ag_1_/ZSM‐5 SAC and Cu_1_/ZSM‐5 SAC under the same reaction condition as shown in Figure 3a.

The formation of surface hydroxyl radical (⋅OH*) is one of the reasons for the high activity of Ag_1_−Cu_1_/ZSM‐5 hetero‐SAC. It is found that the activation free energy of breaking the first C−H bond of methane by the non‐bridge hydroxyl (0.81 eV) is 0.24 eV lower than that on the bridged hydroxyl (1.05 eV) as shown in Figure S22 (Supporting Information). The further Bader charge analysis indicates that the non‐bridge hydroxyl group at Z[Cu(µ‐OH)Ag(OH)]^2+^ (IM4 in Figure 4b) exhibits the radical property, while the bridge hydroxyl group is closer to be OH^−^ (Table S4, Supporting Information). Those results indicate that the non‐bridge hydroxyl with the radical property is more reactive than the bridged hydroxyl for the C−H bond activation.

Furthermore, the calculated desorption free energy of hydroxyl radical (Figure 5c) shows that the surface hydroxyl radical (⋅OH*) is the most difficult one to desorb from Ag_1_−Cu_1_/ZSM‐5 hetero‐SAC (1.39 eV) compared with Ag_1_−Cu_p_/ZSM‐5, Ag_p_/ZSM‐5, Cu_1_/ZSM‐5, and Ag_1_/ZSM‐5 samples. The EPR spectra of ⋅OH clearly indicate that the Ag_1_−Cu_1_/ZSM‐5 hetero‐SAC exhibits the lowest concentration of free ⋅OH in the aqueous solution compared with other reference samples as shown in Figure 5d and Table S6 (Supporting Information), which corroborates the DFT calculation results that the surface ⋅OH* is more stable on Ag_1_−Cu_1_/ZSM‐5 hetero‐SAC compared with the other samples. The further DFT calculation results reveal that the free ⋅OH in the aqueous solution would result in the deep oxidation of methanol compared with the surface reactive ⋅OH* (Table S7, Supporting Information). Hence, the stable surface reactive hydroxyl species over Ag_1_−Cu_1_/ZSM‐5 hetero‐SAC would account for DOM with high activity and selectivity.

The DFT calculation data on the desorption energy of the metal cation centres suggests that the Cu_1_‐Ag_1_ active sites ([Cu(µ‐OH)Ag(OH)]^2+^) possess much higher desorption energy (around 2.5 times higher than that of single Cu/Ag atom site) as shown in Figure S23 (Supporting Information), which confirms the strong interplay between Cu and Ag and correspondingly enhances the stability of the Cu atoms in the active site. The DFT simulation results further reveal the reason why Cu species under the reaction conditions are stable. Those DFT results corroborate the experimental results that the Ag and Cu are prone to aggregating in the reaction process of DOM over Ag_1_/ZSM‐5 SAC and Cu_1_/ZSM‐5 SAC. Those results indicate that the sole Ag active sites are less stable and are easily leached into solution during the reaction process of DOM (Table S8, Supporting Information) over Ag_1_/ZSM‐5 SAC. The stability experiments confirm that the productivity of C1 oxygenates from Ag_1_/ZSM‐5 SAC drops dramatically by 90.3% after five cycles (Figure S24, Supporting Information), which corroborates the above DFT results. Those results suggest that the instability of Ag species in Z[Ag(OH)]^+^ sites significantly inhibits the overall catalytic performance of the Ag species as the sole active site (Figure 3a) although the Ag_1_/ZSM‐5 SAC possesses a good initial activity (Figure S25, Supporting Information). Alternatively, the productivity of C1 oxygenates over Ag_1_−Cu_1_/ZSM‐5 hetero‐SAC only drops by 11.1% after five cycles as shown in Figure S24 (Supporting Information). Moreover, the atomically dispersed Cu and Ag species are still stably present and there are no nano‐particles or clusters observed in the used Ag_1_−Cu_1_/ZSM‐5 hetero‐SAC as confirmed by the HAADF‐STEM images (Figure S4, Supporting Information), which corroborates the DFT calculation results that Ag_1_−Cu_1_ dual sites are more stable. Furthermore, the actual Ag and Cu loadings of used Ag_1_−Cu_1_/ZSM‐5 hetero‐SAC after cycles 1 and 5 are almost unchanged (Table S9, Supporting Information), which suggests that there is no significant Ag and Cu leaching during the reaction and further corroborates the DFT calculation results that Ag_1_−Cu_1_ dual sites are more stable. These results reveal that synergistic interaction between silver and copper dual single atoms enables to significantly enhance the stability of active sites of Ag_1_−Cu_1_/ZSM‐5 hetero‐SAC, which corroborates the experimental results of stability testing.

## 3 Conclusion 

In summary, we have designed and constructed ZSM‐5 supported Cu and Ag dual single atoms as a proof‐of‐concept catalyst to demonstrate the unique catalytic performance of hetero‐SACs for converting DOM to high‐value oxygenates by H_2_O_2_. The synthesized Ag_1_−Cu_1_/ZSM‐5 hetero‐SAC yields a methanol productivity of 20115 µmol g_cat_^−1^ and a methanol selectivity of 81% in all products at 70 °C within 30 min and good stability (at least five cycles), superior to most of the state‐of‐the‐art noble and non‐noble metal catalysts in the open literature. Synergistic interaction between silver and copper dual single atoms enables the C−H bond of CH_4_ and the O−O bond of H_2_O_2_ during the DOM process, which correspondingly enhances the catalytic activity, selectivity, and stability. Although further refinement in optimizing the reaction conditions and other factors may improve the catalytic performance, our atomic‐level design strategy on dual‐single‐atom active site should pave the way to designing advanced catalysts for methane conversion.

## 4 Experimental Section

### Synthesis of Catalysts

The recently developed modified adsorption methods were used to prepare the hetero‐SACs of H‐ZSM‐5 supported Cu and Ag dual single atoms via finely tuning the adsorption parameters in the aqueous solution.^[^ ^25^, ^56^, ^57^ ^]^ Typically, H‐ZSM‐5 (SiO_2_/Al_2_O_3_ = 25, the BET surface area of ≈310 m^2^ g^−1^; Tianjin Yuanli Chemical Co.) was dispersed in deionized water (18.2 MΩ cm^−1^). The pH of Ag and Cu precursor (AgNO_3_ and Cu(NO_3_)_2_·3H_2_O; Sinopharm Chemical Reagent Co., China) was finely tuned from 3.5 to 4.0. The diluted Ag and Cu precursors were pumped into H‐ZSM‐5 suspension at a speed of ≈0.5 mL min^−1^ under stirring. After ageing for 2 h at room temperature, the sample was centrifuged, washed and then dried at 60 °C in an oven overnight and calcined at 550 °C for 3 h in static air. The actual loadings of Ag (0.0047 wt%) and Cu (1.26 wt%) were measured by the inductively coupled plasma optical emission spectroscopy (ICP‐OES) method (Table S5, Supporting Information). The other Pd_1_‐Cu_1_/ZSM‐5 hetero‐SAC, Ir_1_‐Cu_1_/ZSM‐5 hetero‐SAC, Pt_1_‐Cu_1_/ZSM‐5 hetero‐SAC, Rh_1_‐Cu_1_/ZSM‐5 hetero‐SAC, Au_1_‐Cu_1_/ZSM‐5 hetero‐SAC, Ag_1_/ZSM‐5 SAC, and Cu_1_/ZSM‐5 SAC were prepared in a similar manner by using Pd(NO_3_)_2_, IrCl_3_, H_2_PtCl_6_, Rh(NO_3_)_3_, HAuCl_4_, AgNO_3_, and Cu(NO_3_)_2_ (Sinopharm Chemical Reagent Co., China) as metal precursors. The actual loadings of M (M represents Pd, Ir, Pt, Rh, Au, and Ag) and Cu were measured by the ICP‐OES method and listed in Table S5 (Supporting Information). The dispersion status of Ag and Cu species are thoroughly characterized by HAADF‐STEM, EXAFS, EPR, and in situ NO‐DRIFTs, which detailedly discussed in the Results and Discussion Section.

The Ag and Cu nanoparticle catalyst (Ag_p_−Cu_p_/ZSM‐5) was prepared by deposition–precipitation.^[^ ^58^ ^]^ Typically, H‐ZSM‐5 was dispersed in 100 mL deionized water, tuning the pH of the solution to 7.0 by NaOH (purchased from Aladdin) and taking corresponding content AgNO_3_ and Cu(NO_3_)_2_·3H_2_O in 20 mL deionized water to prepare metal precursor solution. Next, the diluted Ag and Cu precursors were pumped into H‐ZSM‐5 suspension at a speed of ≈0.5 mL min^−1^ under stirring. At the same time, the solution of H‐ZSM‐5 was heated to 80 °C. After ageing for 2 h at 80 °C, the sample was centrifuged and washed, finally dried at 60 °C in an oven overnight and calcined at 550 °C for 3 h in 5% H_2_. The actual loadings of Ag (0.0052 wt%) and Cu (1.29 wt%) were measured by the ICP‐OES (Table S10, Supporting Information). The same synthesis method was also used to prepare Ag_p_/ZSM‐5 and Cu_p_/ZSM‐5 via using AgNO_3_ or Cu(NO_3_)_2_·3H_2_O as the sole metal precursor.

Ag single atom with Cu nanoparticles supported on ZSM‐5 (Ag_1_−Cu_p_/ZSM‐5) was firstly loaded with Cu nanoparticles using a deposition–precipitation method, and then Ag single atoms were loaded via a modified adsorption method.^[^ ^25^, ^56^, ^57^ ^]^ The actual loadings of Ag (0.0043 wt%) and Cu (1.11 wt%) were measured by the ICP‐OES (Table S10, Supporting Information).

### Evaluation of Catalytic Performance

DOM was performed in a 210 mL Teflon‐coated stainless steel autoclave. The catalyst (typically 22 mg) was dispersed in 21.05 mL of 0.489 m H_2_O_2_ (Sinopharm Chemical Reagent Co. China) aqueous solutions. The charged autoclave was sealed and purged three times with CH_4_ gas. It was then pressurized to a desired pressure (typically 30 bar) with CH_4_ gas. The solution was heated to desired reaction temperature (typically 70 °C), where a thermocouple was directly inserted into the solution to measure the temperature. Once the temperature reached the set value, the solution was vigorously stirred at ≈1200 rpm for a certain time (typically 30 min). After the reaction, the autoclave was cooled in an ice‐water mixture to minimize the loss of volatile products. The reactor was connected with gas chromatography (GC), and then the gaseous composition was analyzed by GC (PT column) equipped with a methanizer unit and FID detector. Only CO_2_ and CH_4_ could be detected in a typical GC of the gas mixture after DOM (Figure S26, Supporting Information). Oxygenates in the liquid were analyzed by ^1^H NMR spectra on a Bruker 400 MHz NMR, 3‐(trimethylsilyl)‐1‐propanesulfonic acid sodium salt (DSS, purchased from TCI) was used as a calibration standard. The solvent suppression technique was run to suppress the dominant H_2_O signal during NMR measurement. Typically, 0.7 mL of product solution was mixed with 0.1 mL D_2_O (with 0.1012 µmol DSS) in the tube. The ^1^H NMR spectra of liquid products over Ag_1_/ZSM‐5 SAC and Ag_1_−Cu_1_/ZSM‐5 hetero‐SAC after DOM were shown in Figure S27 (Supporting Information). The identified oxygenated products in liquid were methanol (*δ* = 3.34 ppm), methyl hydroperoxide (*δ* = 3.86 ppm), methanediol (*δ* = 5.04 ppm), and formic acid (*δ* = 8.28 ppm). To quantify the products accurately, their standard curves were established respectively (Figure S28, Supporting Information). Methanediol (CH_2_(OH)_2_), a product of the hydration of formaldehyde, was obtained from the diluted commercial aqueous solution of formaldehyde. Methyl hydroperoxide (CH_3_OOH) was prepared in the lab by using the method reported by Davies and co‐workers.^[^ ^59^ ^]^

The amount of products was calculated by using their standard curves. The detailed calculation method is as follows: the peak area ratio of product to standard substance (DSS) is obtained by the ^1^H NMR spectrum. The ratio of product to standard substance (DSS) in the tube is obtained by bringing it into the standard curve. Multiplying the quantity ratio of the above substances by the quantity of DSS substances in the tube (0.1012 µmol) to obtain the amount of the product in the tube. Finally, the amount of product in the solution after DOM is obtained by multiplying the corresponding proportional coefficient.

The mass yields of catalysts and selectivity of methanol were calculated by using the following equations:

(1) $$\mathrm{Yields}\;\mathrm{of}\;\mathrm{product}\;\left(\mu \;\mathrm{mol}\;g_{\mathrm{cat}}^{-1}\right)=\frac{\mathrm{Products}\;\left(\mu \mathrm{mol}\right)}{\mathrm{Catalyst}\;\left(g\right)}$$

(2) $$\mathrm{Selectivity}\;\mathrm{of}\;\mathrm{methanol}\;\left(\%\right)=\frac{{\mathrm{CH}}_{3}\mathrm{OH}\;\left(\mu \mathrm{mol}\right)}{\mathrm{Total}\;\mathrm{products}\;\left(\mu \;\mathrm{mol}\right)}$$

In the cycling tests, 22 mg catalysts from a total of 222 mg were used for catalytic methane oxidation. After each cycling measurement reaction, a parallel experiment was conducted using the rest of the catalysts under identical reaction conditions. After that, all the catalysts were mixed, washed, collected, and dried at 60 °C for 12 h to remove any organic chemicals adsorbed on catalysts. Then 22 mg of catalysts were used from the collected samples for the next cycling experiment until a total of five cycles.

### Theoretical Calculation Methods and Model Construction

To explain the mechanism of methane oxidation to methanol over Ag_1_−Cu_1_/ZSM‐5 hetero‐SAC, the periodic density functional theory calculations were performed using Vienna Ab‐initio Simulation Package (VASP).^[^ ^60^, ^61^ ^]^ The projector augmented wave (PAW) method was used to describe the interaction between atomic nucleus and electrons. The cutoff energy for the plane wave was set as 450 eV.^[^ ^62^ ^]^ The generalized gradient approximation Perdew–Burke–Ernzerhof (PBE) functional was adopted to describe the exchange correlation.^[^ ^63^ ^]^ The key Van der Waals interaction for molecular sieves was implemented by the DFT‐D3(BJ) method.^[^ ^64^, ^65^ ^]^ The convergence criteria for geometry optimization was 0.05 eV Å^−1^ for the maximum force of all the relaxed atoms. The transition state was searched using the constraint minimization method with the same force convergence criterion.^[^ ^66^ ^]^ It was reported by different groups that the molecular entropy physisorbed in the ZSM‐5 zeolite pore would be lowered by 38% with reference to the gas phase state.^[^ ^67^, ^68^ ^]^ Accordingly, the entropy of physisorbed small molecules in ZSM‐5 zeolite was corrected by this value.

The optimized lattice parameters of ZSM‐5 zeolite are *a* = 20.530 Å, *b* = 20.306 Å, and *c* = 13.629 Å, which is consistent with the experimental data.^[^ ^69^ ^]^ Lonsinger and his collaborators^[^ ^70^ ^]^ found that T12 is the most stable single Al substitution site. Hutchings and co‐workers^[^ ^71^ ^]^ reported that dinuclear species could be anchored on 8 MR. Pidko's group^[^ ^72^ ^]^ also showed that dinuclear metal clusters loaded at T7 and T12 sites on 8 MR could exhibit high activity and stability. Hence, the Si^4+^ at T12 and T7 sites located in the sinusoidal channel *γ*−8MR of ZSM‐5 zeolite were substituted with Al^3+^ (Figure S9, Supporting Information) in the framework for the anchored metal sub‐nanoclusters. The active site was constructed on the basis of the UV–Vis spectrum (Figure S6, Supporting Information) and the EXAFS results (Table S2, Supporting Information).

## Conflict of Interest

The authors declare no conflict of interest.

## Author Contributions

B.Y., L.C., and S.D. contributed equally to this work. B.Y. performed data curation, investigation, formal analysis, and wrote the original draft. L.C. performed DFT data curation, investigation, formal analysis, and wrote the original draft. S.D. performed STEM data curation and formal analysis. Y.J. performed formal analysis. B.Y. performed STEM data curation, and formal analysis. H.L. performed formal analysis. Y.Z. performed investigation. J.X. performed formal analysis. Y.Z. performed investigation. C.P. performed formal analysis. X.C. performed DFT data curation, performed funding acquisition, and supervision, and also reviewed and edited the work. Y.Z. performed funding acquisition and supervision. Y.L. performed conceptualization, methodology, data curation, funding acquisition, wrote the original draft, and reviewed and edited the work.

## Supporting information

Supporting Information

## Data Availability

The data that support the findings of this study are available from the corresponding author upon reasonable request.
