# Supplementary material for: Silver and Copper Dual Single Atoms Boosting Direct Oxidation of Methane to Methanol via Synergistic Catalysis
Source: Adv Sci (Weinh). 2023 Jul 3;10(26):2302143. doi: 10.1002/advs.202302143 (PMC10502841; doi:10.1002/advs.202302143)
Supplement: Supplementary file 1 — Supporting Information [file ADVS-10-2302143-s001.pdf]

ADVANCED SCIENCE
Open Access

Supporting Information

for *Adv. Sci.*, DOI 10.1002/advs.202302143

Silver and Copper Dual Single Atoms Boosting Direct Oxidation of Methane to Methanol via Synergistic Catalysis

Baiyang Yu, Lu Cheng, Sheng Dai, Yongjun Jiang, Bing Yang, Hong Li, Yi Zhao, Jing Xu, Ying Zhang, Chengsi Pan, Xiao-Ming Cao, Yongfa Zhu and Yang Lou**

Supplementary Materials

Silver and Copper Dual Single Atoms Boosting Direct Oxidation of Methane to Methanol via Synergistic Catalysis

Baiyang Yu[a, b#], Lu Cheng[c, d#], Sheng Dai[c#], Yongjun Jiang[c], Bing Yang[e], Hong Li[e], Yi Zhao[a, b], Jing Xu[f], Ying Zhang[a, b], Chengsi Pan[a, b], Xiao-Ming Cao[c, d*], Yongfa Zhu[g], Yang Lou[a, b*]

[a]*Key Laboratory of Synthetic and Biological Colloids, Ministry of Education, School of Chemical and Material Engineering, Jiangnan University, Wuxi, Jiangsu 214122, China*

[b]*International Joint Research Center for Photoresponsive Molecules and Materials, Jiangnan University, Wuxi, Jiangsu 214122, China*

[c]*Key Laboratory for Advanced Materials and Feringa Nobel Prize Scientist Joint Research Center, School of Chemistry and Molecular Engineering, East China University of Science and Technology, Shanghai, 200237, China*

[d]*Centre for Computational Chemistry and Research Institute of Industrial Catalysis, East China University of Science and Technology, Shanghai, 200237, China*

[e]*Dalian National Laboratory for Clean Energy, Dalian Institute of Chemical Physics, 457 Zhongshan Road, Dalian, 116023, China*

[f]*School of Food Science and Technology, Jiangnan University, Wuxi, Jiangsu 214122, China*

[g]*Department of Chemistry, Tsinghua University, Beijing 100084, China*

Corresponding authors: xmcao@ecust.edu.cn; yang.lou@jiangnan.edu.cn.

[#]*Those authors made equal contribution.*

Table of Contents

Catalyst characterization

Figure S1. High-magnification HAADF-STEM images of Ag_p-Cu_p/ZSM-5.

Figure S2. Low-magnification and high-magnification HADDF-STEM images of fresh Ag_1-Cu_1/ZSM-5 hetero-SAC.

Figure S3. Atomic resolution aberration-corrected HAADF-STEM image of Ag_1-Cu_1/ZSM-5 hetero-SAC and the intensity profile of Ag-Cu atoms.

Figure S4. High-magnification and atomic resolution HADDF-STEM images of used Ag_1-Cu_1/ZSM-5 hetero-SAC.

Figure S5. EPR of Ag_1-Cu_1/ZSM-5 hetero-SAC and Ag_p-Cu_p/ZSM-5.

Figure S6. UV-Vis DR spectra of the different catalysts.

Figure S7. XRD pattern profiles of Ag_1-Cu_1/ZSM-5 hetero-SAC, Ag_1-Cu_p/ZSM-5, Ag_p-Cu_p/ZSM-5 and H-ZSM-5.

Figure S8. Wavelet transform (WT) analysis of the Cu K-edge EXAFS oscillations of Ag_1-Cu_1/ZSM-5 hetero-SAC (a), Cu foil (b), Cu_2O (c), and CuO (d).

Figure S9. Model construction of Ag_1-Cu_1/ZSM-5 hetero-SAC.

Figure S10. EXAFS fitting curve for Ag_1-Cu_1/ZSM-5 hetero-SAC by using the model of hydrated copper ion.

Figure S11. First derivative of copper K edge X-ray absorption near-edge structure spectra over Ag_1-Cu_1/ZSM-5 hetero-SAC.

Figure S12. The Mayer bond valence analysis of $Z[Cu(OH)]^+[Ag(OH)]^+$.

Figure S13. C1 yields and selectivity of methanol on half Ag_1/ZSM-5 SAC-half Cu_1/ZSM-5 SAC and Ag_1-Cu_1/ZSM-5 hetero-SAC.

Figure S14. Reaction kinetics of direct selective oxidation of methane to methanol of different dispersion catalysts.

Figure S15. The activation free energies of the first C-H bond breaking of methane at Ag_1-Cu_1/ZSM-5 and Pd_1-Cu_1/ZSM-5.

Figure S16. Changing the content of silver and reaction condition for DOM on Ag_1-Cu_1/ZSM-5 hetero-SAC.

Figure S17. Different DFT models established through similar coordination environment or valence states.

Figure S18. The relative energy distributions of possible aluminium pairs (-[AlO-$(Si\text{-}O)_n$-AlO]-) of H-ZSM-5.

Figure S19. $Z[Cu(\mu\text{-}O)Ag(H_2O)]^{2+}$ (IM3) site (a) and the activation free energies of the first C-H bond breaking of methane and the first O-H bond breaking of water at $Z[Cu(\mu\text{-}O)Ag(H_2O)]^{2+}$ (IM3) site (b).

Figure S20. ^{1}H NMR spectrums of liquid mixtures over Ag_1-Cu_1/ZSM-5 hetero-SAC after DOM.

Figure S21. The first C-H bond activation free energy of methane at different sites.

Figure S22. The activation free energies of the first C-H bond breaking of methane by the non-bridge hydroxyl and bridge hydroxyl of $Z[Cu(\mu\text{-}OH)Ag(OH)]^{2+}$.

Figure S23. The desorption energies of active sites $[Cu(\mu\text{-}OH)Ag(OH)]^{2+}$, $[Ag]^+$, $[Ag(OH)_2]^+$, $[Ag(OH)]^+$, $[Cu]^+$, $[Cu(OH)_2]^+$, and $[Cu(OH)]^+$anchored over the ZSM-5 zeolite framework.

Figure S24. Reaction cycles of DOM coupling on Ag_1/ZSM-5 SAC and Ag_1-Cu_1/ZSM-5 hetero-SAC.

Figure S25. Intrinsic activity of pure Ag_1/ZSM-5 SAC, Cu_1/ZSM-5 SAC and Ag_1-Cu_1/ZSM-5 hetero-SAC.

Figure S26. A typical chromatogram of gas mixtures over Ag_1-Cu_1/ZSM-5 hetero-SAC after DOM and standard curve for CO_2 quantification.

Figure S27. Typical ^1H NMR spectrums of liquid mixtures over Ag_1/ZSM-5 SAC and Ag_1-Cu_1/ZSM-5 hetero-SAC after DOM.

Figure S28. The standard curves of methanol, methyl hydroperoxide, methanediol, formic acid for calculating liquid product after DOM.

Table S1. Catalytic performance of the most promising catalysts in the direct oxidation of methane reported in literature recently.

Table S2. EXAFS fitting results for Ag_1-Cu_1/ZSM-5 hetero-SAC according to the standard crystal model provided by DFT.

Table S3. The DFT calculation results of Bader charge analysis for Cu species over Ag_1-Cu_1/ZSM-5 hetero-SAC and CuO.

Table S4. Bader charge analysis of oxygen at different sites.

Table S5. Nominal and actual M & Cu loading of different catalysts.

Table S6. Intensity of hydroxyl radicals signal in EPR spectra of Ag_1-Cu_1/ZSM-5 hetero-SAC, Ag_1-Cu_p/ZSM-5 and Ag_p-Cu_p/ZSM-5.

Table S7. The standard free energy of activation and the free energy change of each key elementary step in the micropores of ZSM-5 solution at 343 K.

Table S8. Actual Ag loading of used Ag_1/ZSM-5 SAC.

Table S9. Actual Ag & Cu loading of used Ag_1-Cu_1/ZSM-5 hetero-SAC.

Table S10. Actual Ag & Cu loading of different dispersion catalysts.

Supplemental References

Catalyst characterization

X-ray diffraction (XRD) analysis was carried out on a Bruker D8 focus diffraction spectrometer using Cu Kα radiation with a scanning angle (2 theta) of 10-80 ° at a speed of 2 °/min, operated at 40 kV and 40 mA.

The XAS (X-ray absorption spectroscopy) measurements were recorded at the BL14W1 beamlines at the Shanghai Synchrotron Radiation Facility (SSRF, Shanghai, China) in fluorescence mode. The radiation was monochromatized by a Si (111) double-crystal monochromator. The XANES and EXAFS data were processed and analyzed with ATHENA and ARTEMIS software.

Aberration-corrected scanning transmission electron microscopy (AC-STEM) was performed on a ThermoFisher Themis Z microscope equipped with two aberration correctors under 200 kV. High angle annular dark field (HAADF)-STEM images were recorded using a convergence semi angle of 11 mrad, and inner- and outer collection angles of 59 and 200 mrad, respectively. Energy dispersive X-ray spectroscopy (EDS) was carried out using 4 in-column Super-X detectors.

The metal loadings of the catalysts were determined by inductively coupled plasma optical emission spectroscopy (ICP-OES) on an optima 730 instrument (AGILENT ICP-OES 730).

UV-Visible (UV-Vis) spectra of the catalysts were recorded on UV-3600 plus (SHIMADZU) in the diffuse reflectance (DR) mode at room temperature. The baseline was corrected using $BaSO_4$ as a reference material. Samples were scanned from 200 to 800 nm at a scan rate of 120 nm/min. The intensity of the UV-vis DR spectra was presented in the form of the Kubelka-Munk function.

X-ray photoelectron spectra (XPS) were carried out on a Thermo ESCALAB 250Xi spectrometer using Al-Kα radiation source. The binding energies were calibrated using C1s peak of contaminant carbon (Binding Energy = 284.8 eV) as an internal standard.

5,5-dimethyl-1-pyrroline N-oxide (DMPO) spin-trapping electron paramagnetic resonance (EPR) experiments were conducted on a Bruker Emxplus spectrometer at room temperature. 22 mg catalyst, 0.2 mL DMPO (50 mg/mL) and 21.05 mL 0.489 M H_2O_2 solution were added into a 50 ml brown flask and stirred at 1200 rpm for 2 min.

The diffuse reflectance infrared Fourier transform spectra (DRIFTS) were recorded by a FT-IR spectrometer (Nicolet iS 50, Thermo Scientific) with an MCT detector (cooled by liquid N_2). The in-situ sample chamber is equipped with ZnSe windows. For the DRIFTS of CH_4+H_2O_2 and

CH_4+H_2O, the liquid reactants are introduced into the in-situ chamber via the flowing CH_4 gas. Before introducing the reactive gas, all the samples are preheated at 120 ^oC for 1 hour to remove the possible surface residual adsorbates. Then the sample was heated to the set temperature (70 ^oC), the H_2O_2 (H_2O) was introduced into the cell by the flowing CH_4 gas. Then the DRIFTS of CH_4+H_2O_2 and CH_4+H_2O data are recorded by 64 scans with a resolution of 4 cm^{-1} in K-M (Kubelka-Munk) units.

For NO-DRIFTS, all the samples are preheated at 423 K for 2 hours to remove the possible surface residual adsorbates. The DRIFTs data are acquired by 64 scans with a resolution of 4 cm^{-1} in K-M (Kubelka-Munk) units. After cooling the samples to room temperature (25 ^oC), the background spectrum is first collected. Then NO molecules are introduced to the catalysts in the sample cell until the peak intensity of gaseous NO is stable, indicating the NO adsorption is saturated on the surface of catalysts. Pure He is used to remove the gaseous NO from the sample chamber in order to observe the chemically bonded NO species. The overall processes are recorded by MCT detector (cooled by liquid N_2) including the collection of the background spectrum, probe molecules adsorption and He purging process.

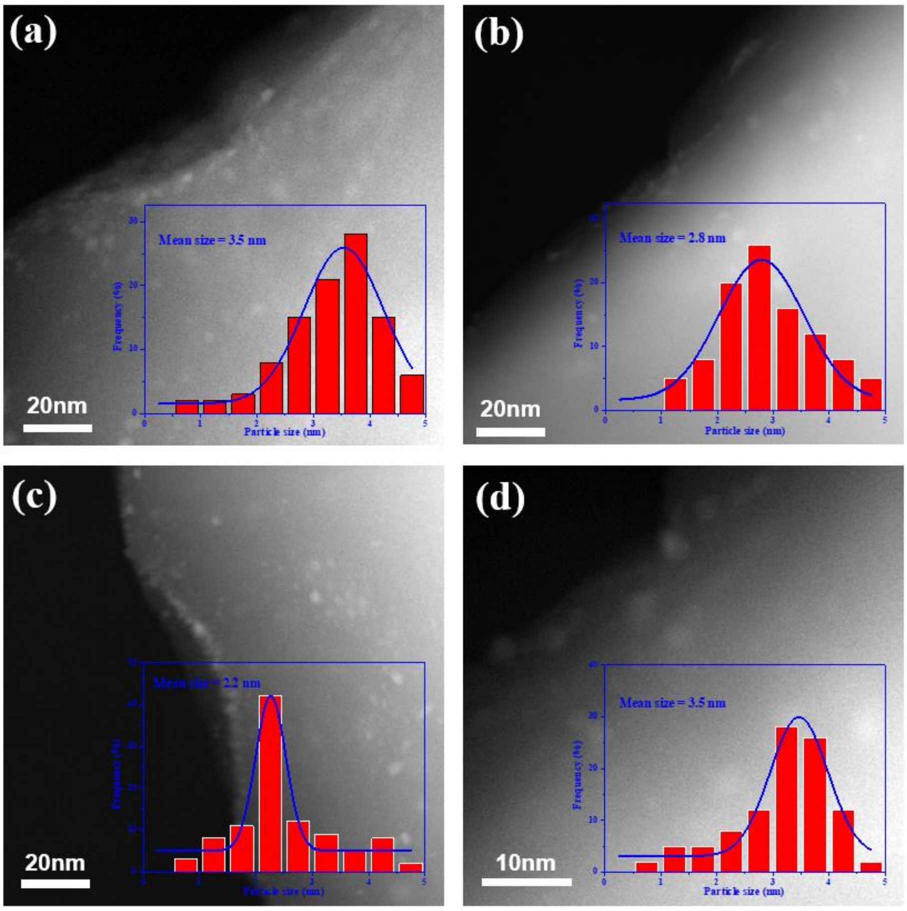

Figure S1. High-magnification HAADF-STEM images of Ag_p-Cu_p/ZSM-5.

The data is acquired by using the software to count the number and the size of nano particles. Particles of Ag_p-Cu_p/ZSM-5 are distributed on the surface of ZSM-5 and the statistical sizes are 3.0 ± 0.8 nm (particle size distribution curve embedded in figures a-d).

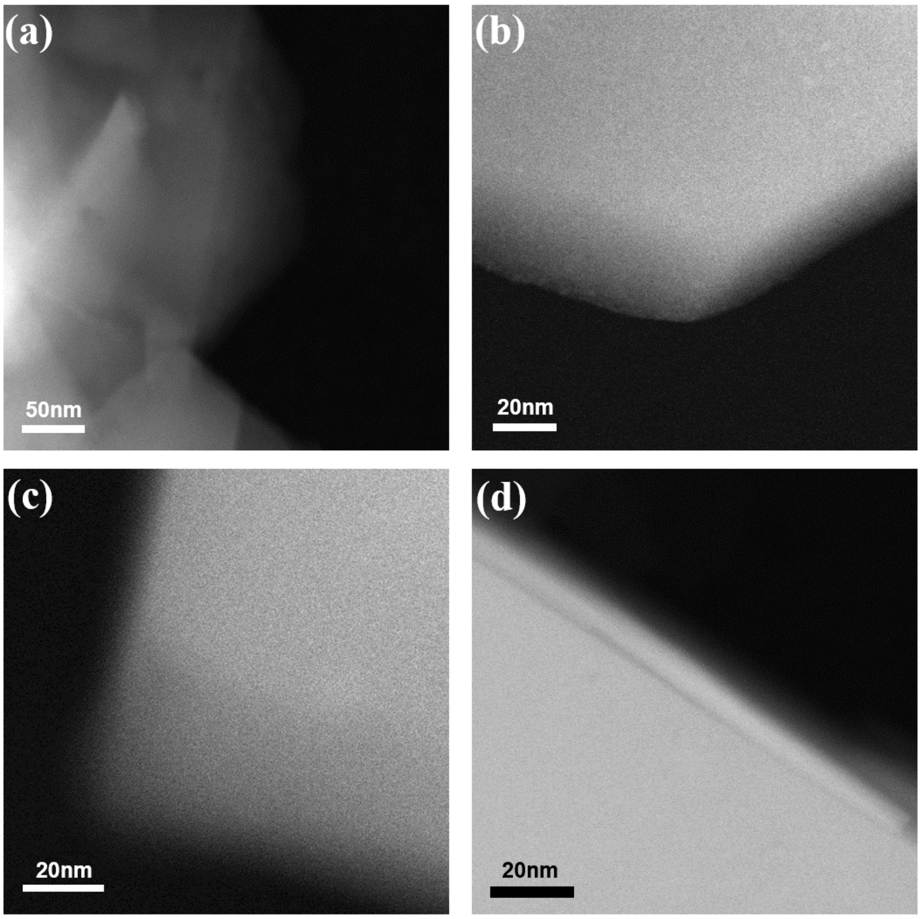

Figure S2. Low-magnification and high-magnification HADDF-STEM images of fresh Ag_1-Cu_1/ZSM-5 hetero-SAC.

As clearly shown in the STEM images, there are no any nano particles in the fresh Ag_1-Cu_1/ZSM-5 hetero-SAC, which indicates silver and copper species are atomically dispersed on H-ZSM-5.

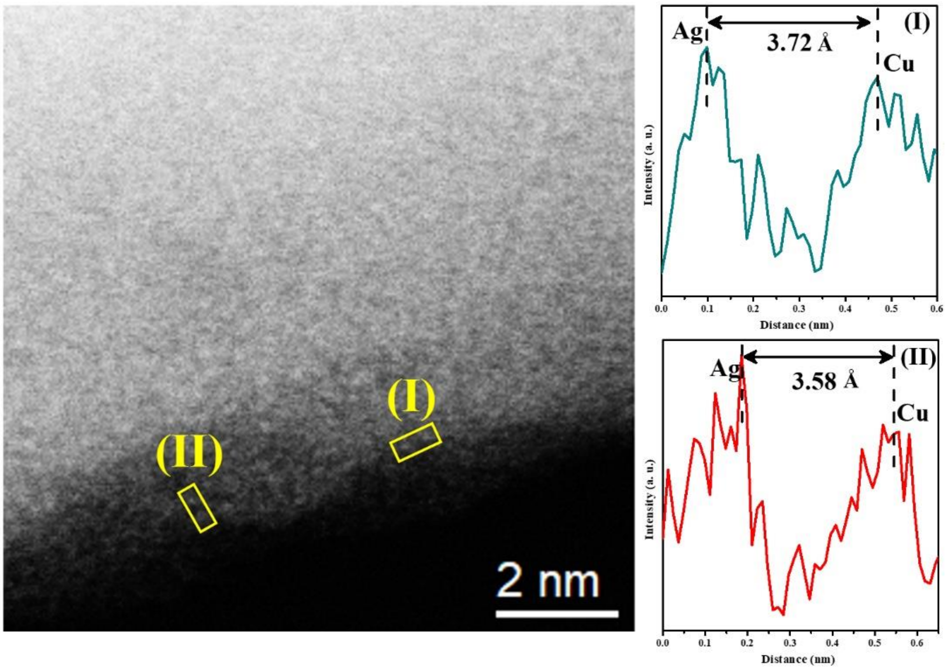

Figure S3. Atomic resolution aberration-corrected HAADF-STEM image of Ag_1-Cu_1/ZSM-5 hetero-SAC and the intensity profile of Ag-Cu atoms.

Due to enhanced Z-contrast, the Ag and Cu atoms can be differentiated under HAADF imaging. Despite some disturbance by the zeolite framework, the intensity profile of the marked sites can still provide a strong indication of adjacent Ag-Cu atomic pairs (average distance of 3.65 Å) by using atomic resolution HAADF-STEM.

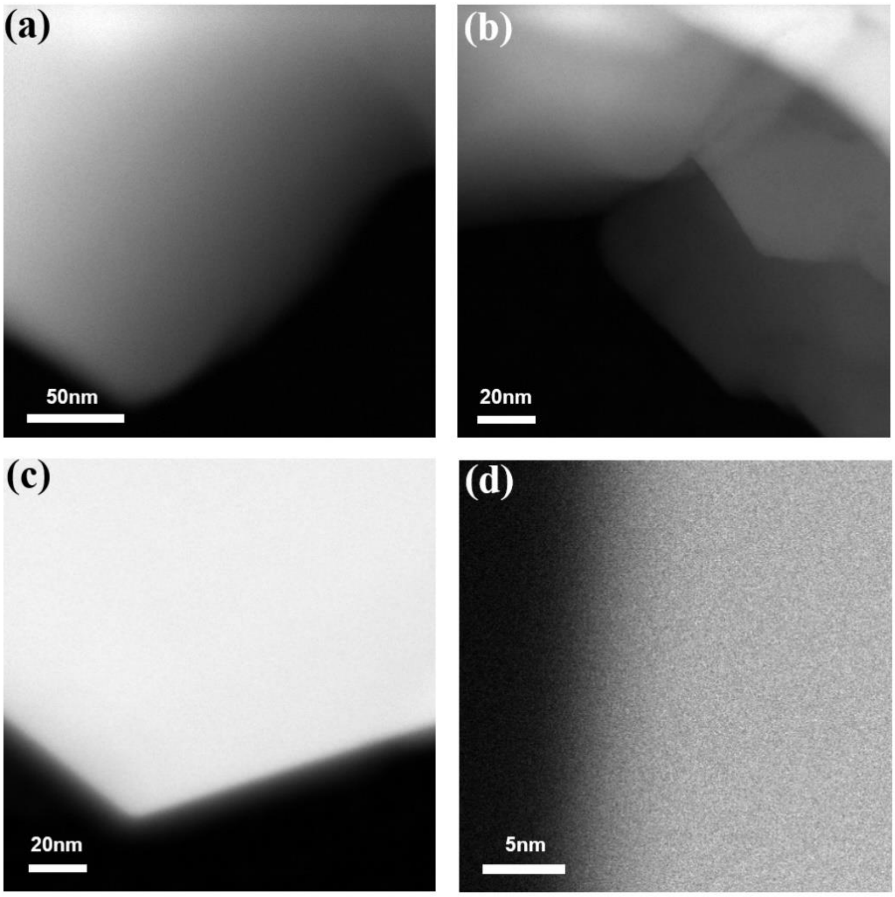

Figure S4. High-magnification and atomic resolution HADDF-STEM images of used Ag_1-Cu_1/ZSM-5 hetero-SAC.

As clearly shown in the HAADF-STEM images, isolated individual Ag/Cu atoms are uniformly dispersed on used Ag_1-Cu_1/ZSM-5 hetero-SAC (30 bar CH_4 for 30 min at 70 ºC, details shown in experimental section). By examining numerous HAADF-STEM images obtained from different regions of the catalyst samples, we unambiguously conclude that silver and copper species are atomically dispersed in the used Ag_1-Cu_1/ZSM-5 hetero-SAC, which suggest that the Ag_1-Cu_1/ZSM-5 hetero-SAC is stable under reaction condition.

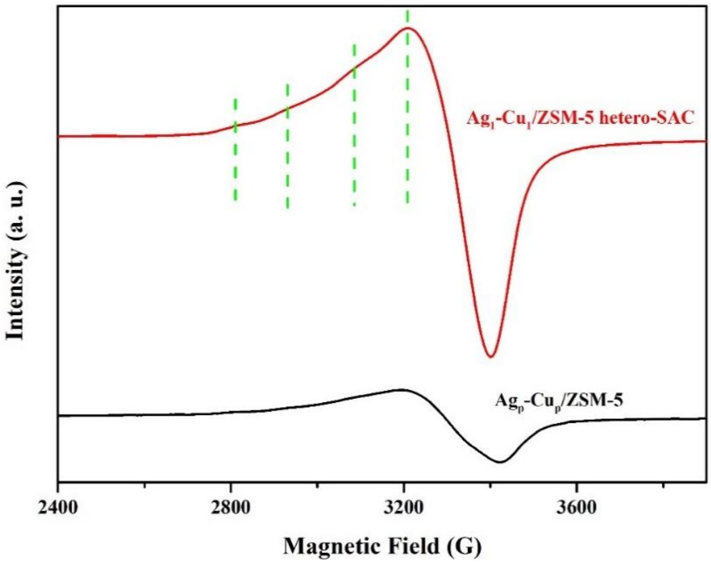

Figure S5. EPR of Ag_1-Cu_1/ZSM-5 hetero-SAC and Ag_p-Cu_p/ZSM-5.

The g components of Ag_1-Cu_1/ZSM-5 hetero-SAC are split into quartets due to the hyperfine interaction between unpaired electrons and copper nucleus, which indicates the interference between neighboring Cu atoms is neglected.[5,6] In other words, each Cu atom is isolated on ZSM-5. Alternatively, the absence of such delicate structure in Cu particles (Ag_p-Cu_p/ZSM-5) indicates the presence of notable interference between neighboring Cu atoms.

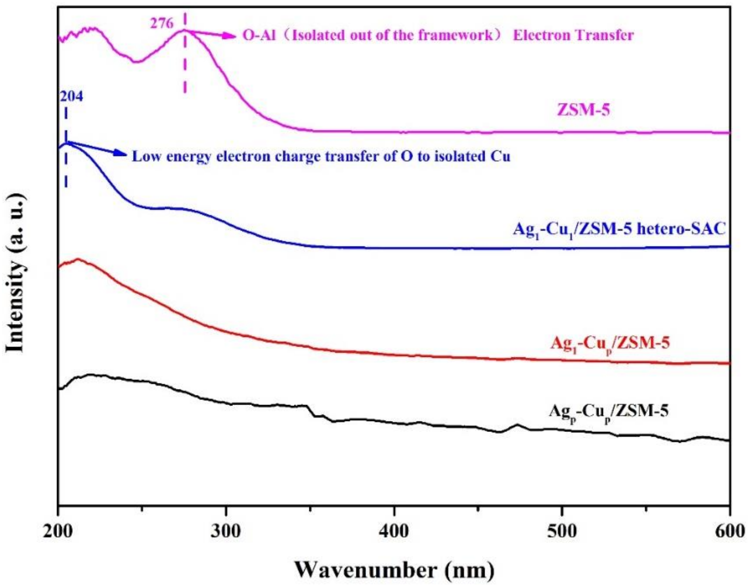

Figure S6. UV-Vis DR spectra of the different catalysts. Scanning wavelength: 200-600 nm. Scanning speed: 2 nm•s^{-1}. All samples were scanned more than three times to eliminate the experimental error.

UV-Vis DR spectra show that the pure ZSM-5 sample possesses a bridge absorption peak at 276 nm, which is associated with the electron transfer from oxygen ligands in the molecular sieve framework to aluminum isolated outside the framework.[1] In the high energy region (200-220 nm), only the silver and copper dual single atoms catalyst has an absorption peak at 204 nm, representing the low-energy charge transition from the oxygen ligand in the zeolite skeleton to the isolated copper single atom.[2-4] The Ag_p-Cu_p/ZSM-5 has no obvious absorption band in the whole UV-Vis absorption band (200-600 nm). UV−Vis DR spectra clearly show that there is an obvious gap between catalysts with different dispersion degrees and pure ZSM-5, which further confirms the conclusion from HAADF-STEM.

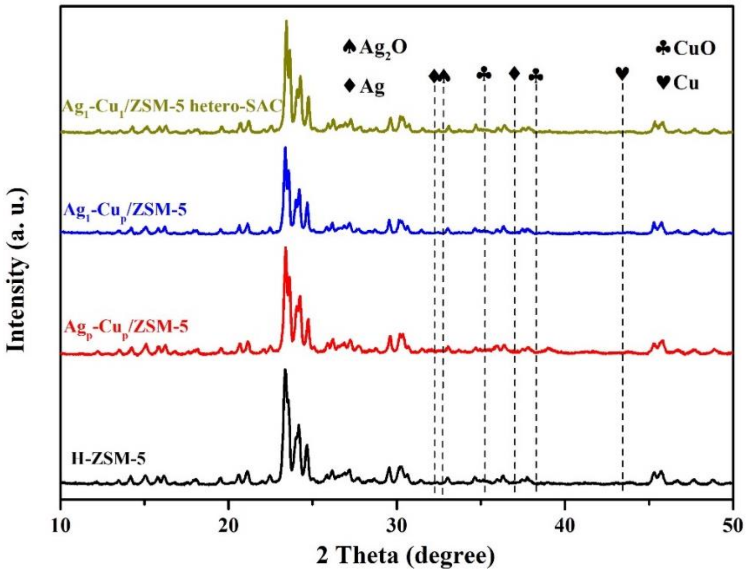

Figure S7. XRD pattern profiles of Ag_1-Cu_1/ZSM-5 hetero-SAC, Ag_1-Cu_p/ZSM-5, Ag_p-Cu_p/ZSM-5 and H-ZSM-5.

There are no any characteristic peaks of Cu (43.3°), CuO particles (35.2 and 38.4°), Ag (32.2 and 37.0°) and Ag_2O (32.7°) that can be observed from XRD patterns of Ag_1-Cu_1/ZSM-5 hetero-SAC, which indicates copper species are highly dispersed on H-ZSM-5.

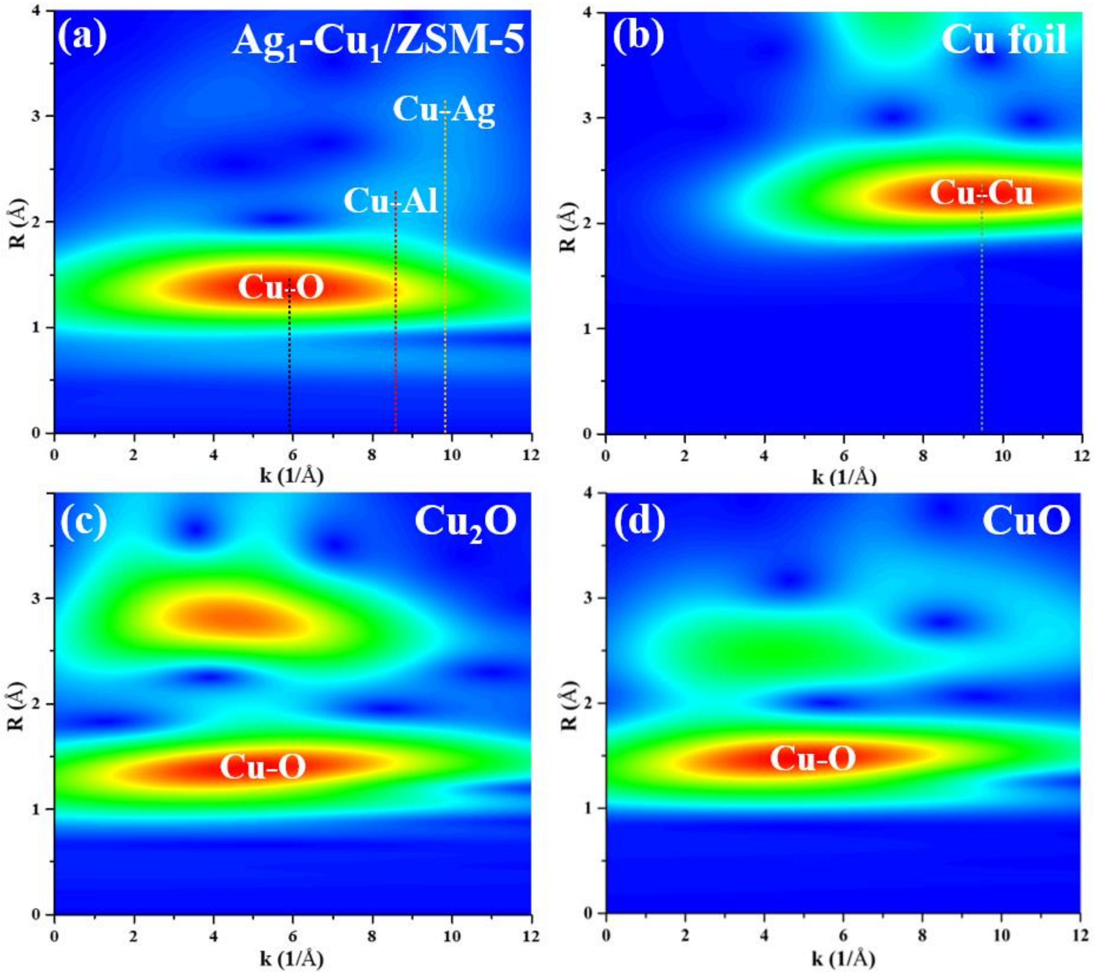

Figure S8. Wavelet transform (WT) analysis of the Cu K-edge EXAFS oscillations of Ag_1-Cu_1/ZSM-5 hetero-SAC (a), Cu foil (b), Cu_2O (c), and CuO (d).

To reveal the atomic dispersion of Cu species in Ag_1-Cu_1/ZSM-5 hetero-SAC more clearly, wavelet transform (WT) of the Cu K-edge EXAFS oscillations is analyzed. The WT contour plots of Ag_1-Cu_1/ZSM-5 hetero-SAC present only one intensity maximum at approximately 6.0 $Å^{-1}$ that can be assigned to the Cu-O coordination and no intensity maximum related to Cu-Cu or Cu-O-Cu coordination can be observed, compared with that of Cu foil, Cu_2O, and CuO references. Meanwhile, there is a weak intensity on Ag_1-Cu_1/ZSM-5 hetero-SAC at approximately 9.8 $Å^{-1}$ that can be assigned to the Ag-Cu scattering and the distance of Y-axis between Ag and Cu atom is about 3.1 Å in WT (around 3.6 Å in real space).[7] Those results confirm that Cu atoms are atomically dispersed on the Ag_1-Cu_1/ZSM-5 hetero-SAC and further indicate that the distance of adjacent Ag-Cu atomic pairs, which is in good agreement with the data of AC-STEM.

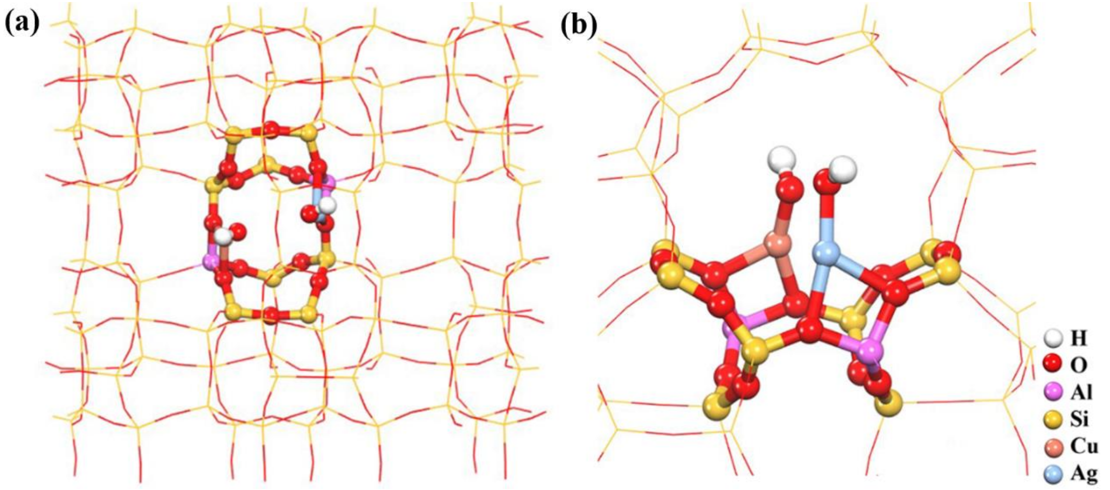

Figure S9. Geometry Structure of Ag_1-Cu_1/ZSM-5 hetero-SAC (a). Only the local optimized structure of γ-8MR/ZSM-5 is shown here (b).

The optimized structure of the Ag_1-Cu_1/ZSM-5 hetero-SAC was constructed according to the UV-Vis spectrum and the EXAFS. In this structure, both the copper and silver ions form three-coordination structures. The local optimization of the active structure (**Figure S9b**) shows that copper and silver single atoms are adsorbed on the γ-8MR of ZSM-5 zeolite.

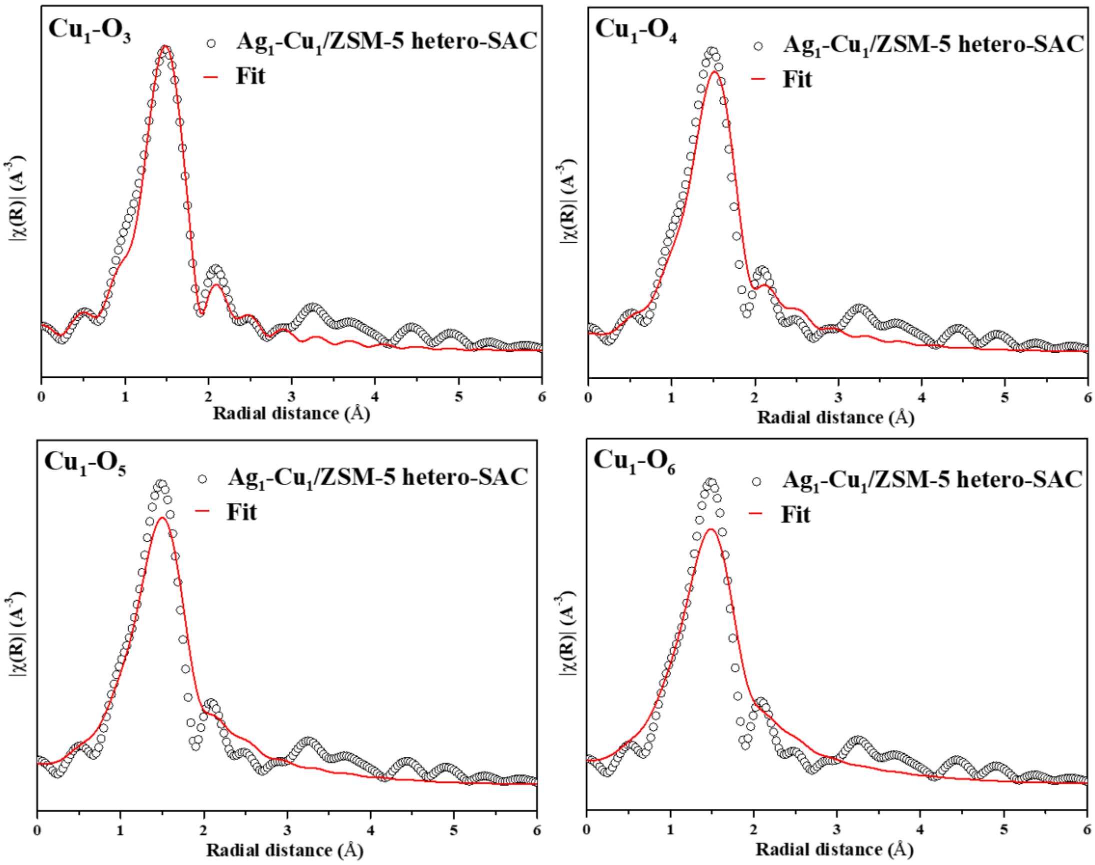

Figure S10. EXAFS fitting curve for Ag_1-Cu_1/ZSM-5 hetero-SAC by using the model of hydrated copper ion.

There is a significant deviation between the fitting curve and the original data when hydrated copper ions are used as the standard model for EXAFS fitting, which further excludes the existence of hydrated copper ions in Ag_1-Cu_1/ZSM-5 hetero-SAC.

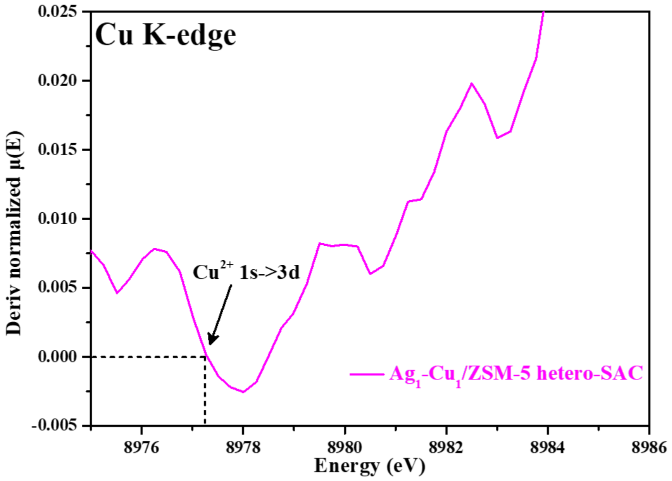

Figure S11. First derivative of copper K edge X-ray absorption near-edge structure spectra over Ag_1-Cu_1/ZSM-5 hetero-SAC.

The first derivative of the Cu K-edge XANES data shows that there is one derivative extremum value found at 8977 eV, which further confirms the Cu species in Ag_1-Cu_1/ZSM-5 hetero-SAC mainly exist as high oxidation state.

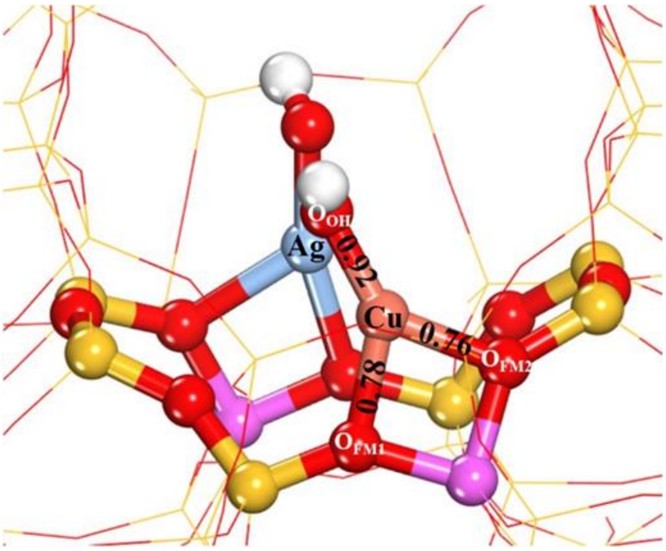

Figure S12. The Mayer bond valence analysis of Z[Cu(OH)]$^+$[Ag(OH)]$^+$. Black number represents Mayer bond order.

The Mayer bond valence analysis was performed to characterize the valence state of Z[Cu(OH)]$^+$[Ag(OH)]$^+$. The Mayer bond valences of Cu-O_{OH}, Cu-O_{FM1}, and Cu-O_{FM2} are 0.92, 0.78, and 0.76, respectively. The formal charge of Cu was also calibrated with reference to the Bader charges of Cu^+ in bulk Cu_2O (+0.55 |e|) and Cu^{2+} in CuO (+1.08 |e|). The calculated Bader charge of Cu at Z[AgOH]$^+$[CuOH]$^+$ is +0.98 |e|. These results confirmed the high oxidation state of Cu in Z[Cu(OH)]$^+$[Ag(OH)]$^+$.

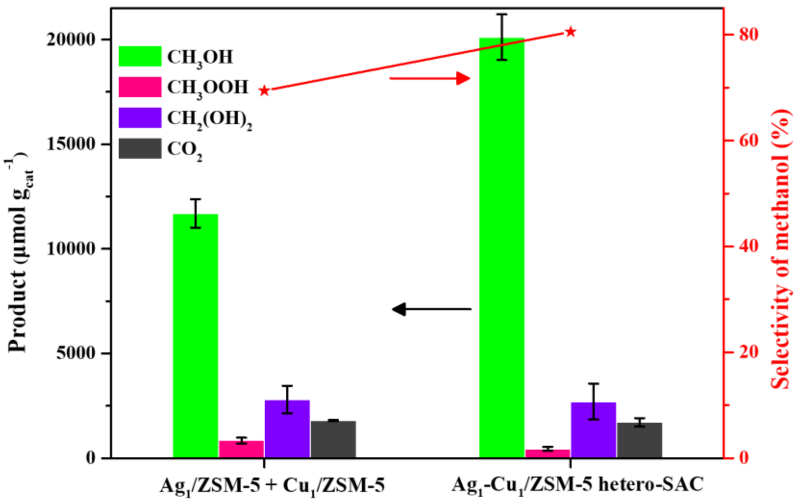

Figure S13. C1 yields and selectivity of methanol on half Ag_1/ZSM-5 SAC-half Cu_1/ZSM-5 SAC and Ag_1-Cu_1/ZSM-5 hetero-SAC. Reaction Condition: 22 mg catalysts, 21.05 ml H_2O_2, 70 ºC, 30 bar CH_4 for 30 min.

The yield of methanol over the physically mixed Ag_1/ZSM-5 and Cu_1/ZSM-5 (50:50 in wt.%) for DOM is 11,700 μmol•g_{cat}^{-1} with a selectivity of 69%, which is significantly lower than that of Ag_1-Cu_1/ZSM-5 hetero-SAC. This result clearly indicates that there is a significant synergistic effect between neighboring Cu and Ag dual single atoms, which not only boosts the catalytic activity but also promotes the selectivity of methane to methanol.

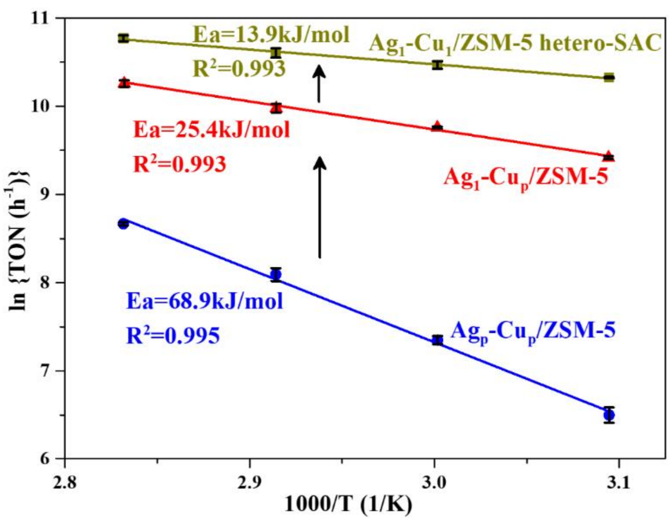

Figure S14. Reaction kinetics of direct selective oxidation of methane to methanol on Ag_1-Cu_1/ZSM-5 hetero-SAC (square symbols), Ag_1-Cu_p/ZSM-5 (triangle symbols) and Ag_p-Cu_p/ZSM-5 (circle symbols) in the temperature (T) range of 50 to 80 °C. Reaction conditions: 22 mg catalysts, 21.05 ml H_2O_2 (0.489 M), 3.0 MPa CH_4 for 30 min with varying reaction temperatures from 50 to 80 °C. All data points were tested for three times to obtain error bar.

The kinetic data confirms that the apparent activation energy on Ag_1-Cu_1/ZSM-5 hetero-SAC (13.9 kJ/mol) is much lower than that on Ag_p-Cu_p/ZSM-5 nano particle (68.9 kJ/mol), and Ag_1-Cu_p/ZSM-5 (25.4 kJ/mol) under the same reaction condition, which indicates that the catalytic process of DOM is intrinsically boosted on hetero-SACs compared with the nano particles.

Turnover number (TON) is defined as:

$$TON = \frac{\text{Yield of methanol } (\mu mol \bullet g_{cat}^{-1})}{\text{Time (h)}} \tag{1}$$

The apparent activation energy (E_a) is calculated by Arrhenius equation:

$$\ln(TON) = -E_a/RT + \ln A \tag{2}$$

E_a is obtained from the slope of the fitting curve.

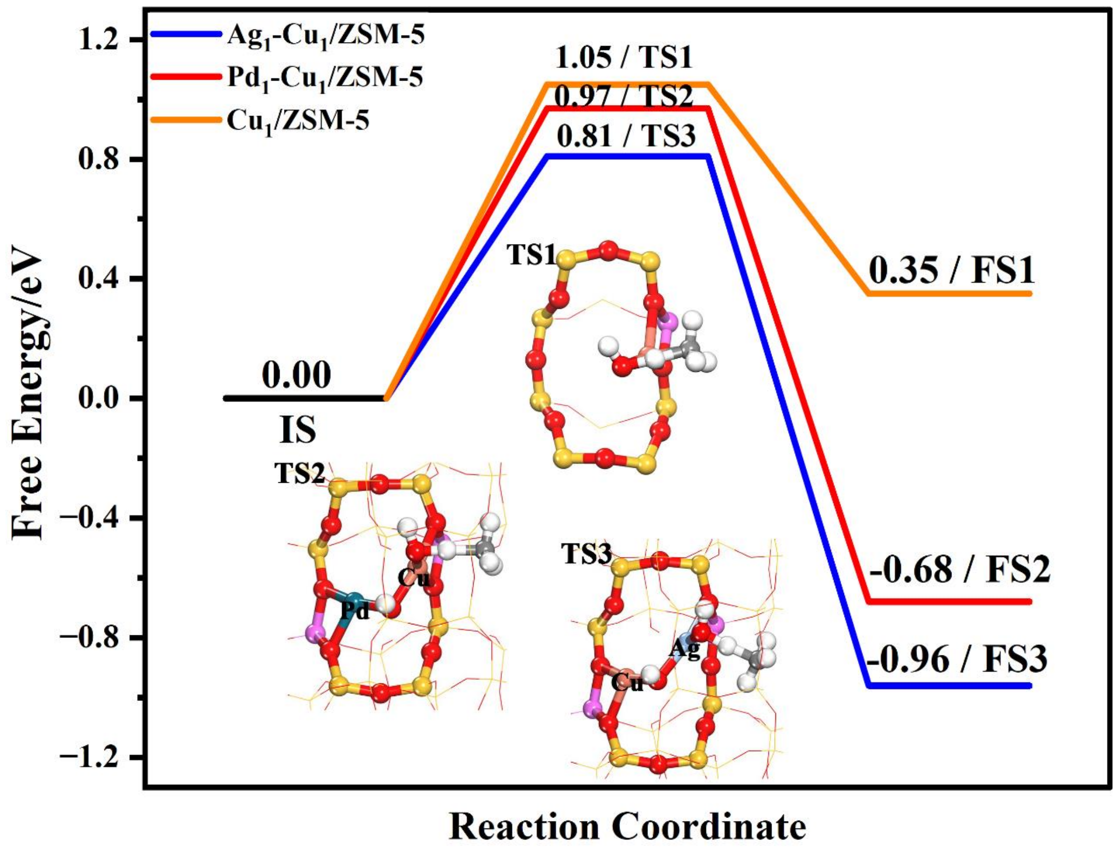

Figure S15. The activation free energies of breaking the first C-H bond of methane at Ag_1-Cu_1/ZSM-5 and Pd_1-Cu_1/ZSM-5.

In this work, the constructed Ag_1-Cu_1/ZSM-5 hetero-SAC exhibits excellent catalytic performance for methane direct oxidation to methanol and other high-value oxygenates. We believe our atomic-level design strategy on dual-single-atom active sites should pave the way to design advanced catalysts for methane conversion. In order to understand the increasing trend of catalyst activity, the activity trend from single atom Cu, dual single atom Pd-Cu to dual single atom Ag-Cu systems for methane C-H bonds activation is computationally investigated by DFT calculations. The active sites are $Z[CuOH]^{+}$, $Z[Pd(\mu\text{-}OH)Cu(OH)]^{2+}$ and $Z[Cu(\mu\text{-}OH)Ag(OH)]^{2+}$, respectively. It is clear that the free energy barriers of the methane C-H bond activation are sequentially lowered in the order of Cu_1/ZSM-5 > Pd_1-Cu_1/ZSM-5 > Ag_1-Cu_1/ZSM-5, which is in line with the trends observed in **Figure 3d** in the main manuscript. Then the transition state structure proves that the active oxygen species that determines the activation of methane C-H bond is non-bridged hydroxyl. The further Bader charge analysis (**Table S4**) indicates that the less negatively charged of the hydroxyl oxygen at the catalyst, the higher reactivity for C-H bond activation. The radical character of non-bridged hydroxyl at Ag_1-Cu_1/ZSM-5 hetero-SAC exhibits the highest intrinsic activity.

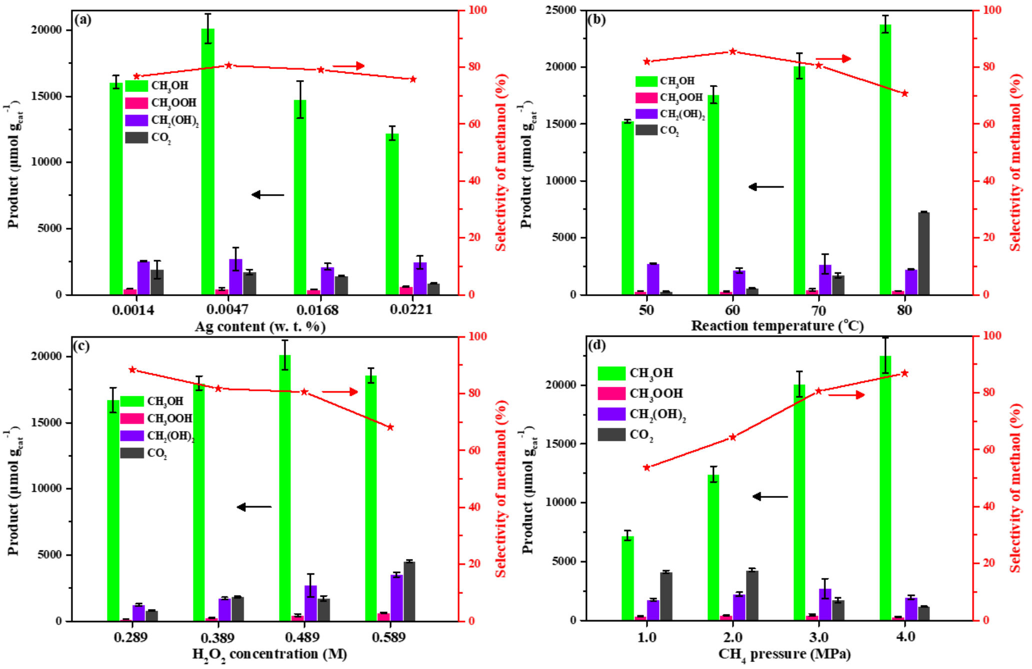

Figure S16. Changing the content of silver and reaction condition for DOM on Ag_1-Cu_1/ZSM-5 hetero-SAC. Productivity with different Ag loading of Ag_1-Cu_1/ZSM-5 hetero-SAC for DOM (a); Productivity of DOM of Ag_1-Cu_1/ZSM-5 hetero-SAC on different temperature (b); concentration of H_2O_2 (c) and methane pressure (d). Reaction Condition (a): 22 mg catalysts, 21.05 ml 0.489 M H_2O_2, 70 ºC, 30 bar CH_4 for 30 min. Reaction Condition (b): 22 mg catalysts, 21.05 ml 0.489 M H_2O_2, 30 bar CH_4 for 30 min. Reaction Condition (c): 22 mg catalysts, 21.05 ml H_2O_2 (from 0.289 M to 0.589 M), 70 ºC, 30 bar CH_4 for 30 min. Reaction Condition (d): 22 mg catalysts, 21.05 ml 0.489 M H_2O_2, 70 ºC, reaction 30 min. Insert, all data points were retested at least three times to obtain error bar.

The most active Ag_1-Cu_1/ZSM-5 hetero-SAC has been selected to optimize the loadings of silver and reaction condition. First, by changing the concentration of silver, we produced Ag_1-Cu_1/ZSM-5 hetero-SAC with different Ag loadings (x=0.0014%, 0.0047%, 0.0168%, and 0.0221%). The silver loading of 0.0047% exhibits the highest activity for DOM. Increasing the silver loading reduces the overall product yield, but the selectivity of methanol does not change obviously. These results further indicate silver species only change the activity of reaction, rather than to alter the conversion direction of DOM. The best Ag loading (0.0047%) of Ag_1-Cu_1/ZSM-5 hetero-SAC has been selected to investigate the effect of reaction temperature on product yield. As the reaction temperatures increase from 50 to 80 ºC, the yield of methanol enhances from 15, 200 to 23, 700 μmol•g_{cat}^{-1} within 30 min while the amount of other C1 products are almost unchanged, suggesting that high temperatures promote the conversion of methane to methanol. But meanwhile, the amount of carbon dioxide (over-oxidation products from DOM) increase exponentially (from 270 to 7,270 μmol•g_{cat}^{-1}), which tremendously reduces the selectivity of methanol. Afterwards, the effects of different hydrogen peroxide concentrations on the product distribution are further investigated. The highest yield of methanol is obtained at a H_2O_2 concentration of 0.489 M. Excessive hydrogen peroxide quickly oxidizes methanol to carbon dioxide, thereby reducing the yield and selectivity of methanol. Moreover, it can be seen that the output of methanol increases proportionally with the increase of methane pressure, indicating high methane pressure benefits the high methanol yield.

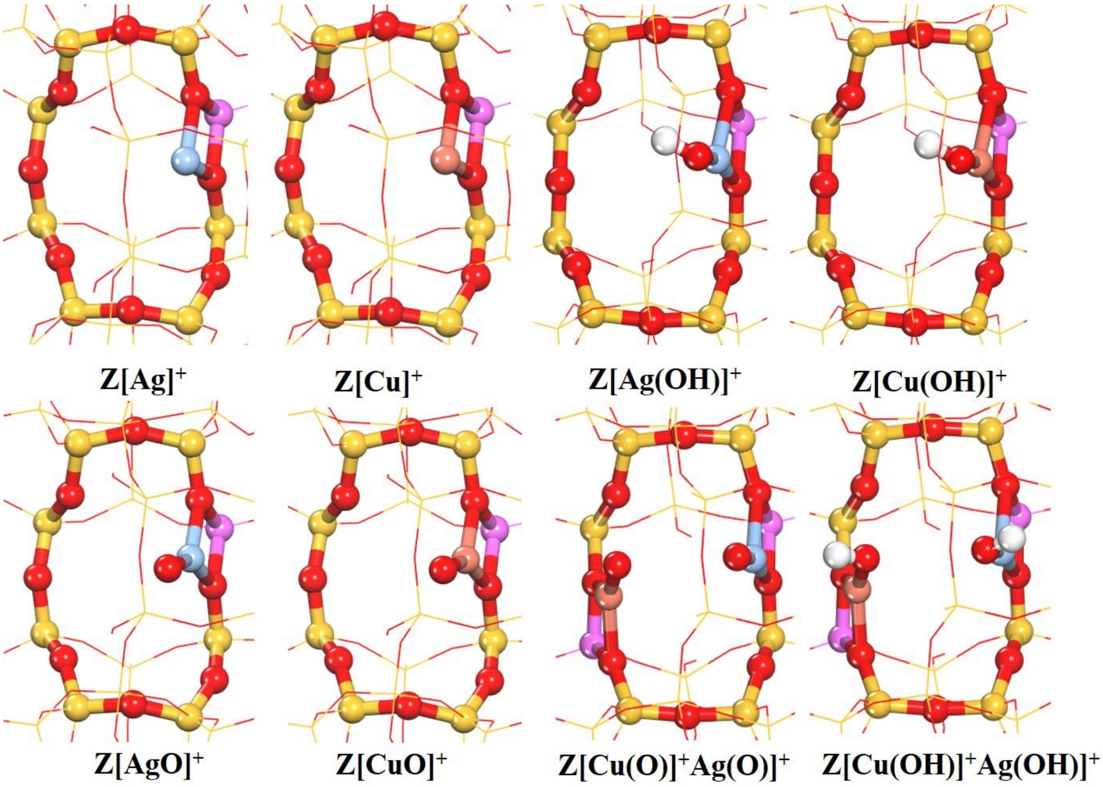

Figure S17. Different DFT models established through similar coordination environment or valence states.

Based on the above characterization data and catalytic behaviors, several model active sites have been searched, but only the Z[Cu(OH)]$^+$[Ag(OH)]$^+$ (IM1 in **Figure 4b** and **Figure S9**) is the most reliable reaction site, where neighboring dual copper and silver single atoms with three-fold coordination are located at the γ-8MR of ZSM-5 zeolite without the bonding between copper and silver single atoms.

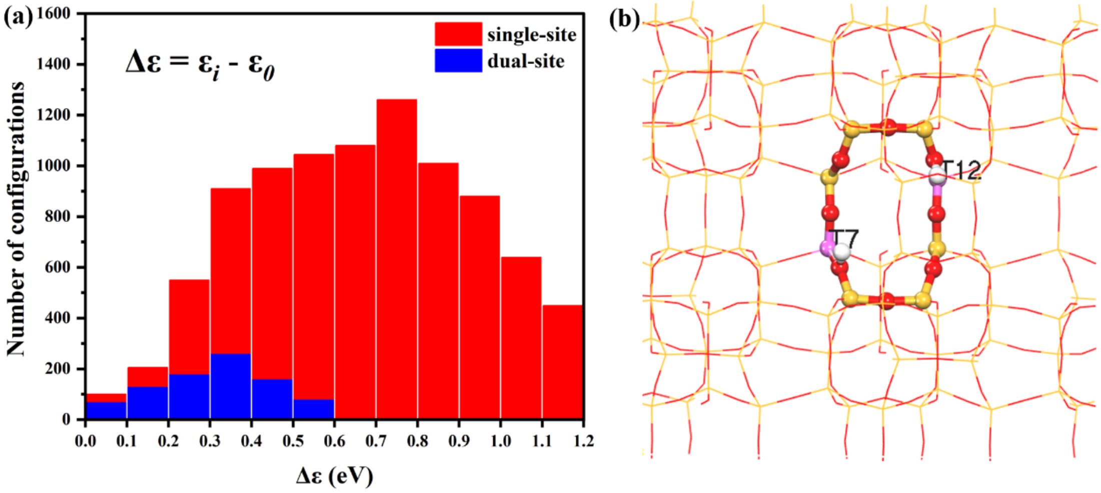

Figure S18. The relative energy distributions of aluminium pairs (-[AlO-(Si-O)$_n$-AlO]-) of H-ZSM-5 were formed (a); The most stable configuration of the two Al exchange sites (b).

We calculated the probability of required two Al cations in the studied H-ZSM-5. The energy distributions of possible aluminium pairs (-[AlO-(Si-O)$_n$-AlO]-) in H-ZSM-5 zeolites were systematically calculated based on DFT calculations, including the aluminium pairs for the formation of both two separated mononuclear sites and dinuclear sites. The energetic distribution of all the possible configuration of aluminium pairs was summarized. The probabilities of the possible configurations were calculated based on Boltzmann distribution:

$$p = \frac{\sum_{i=1}^{m}(n_i * e^{-(\varepsilon_i - \varepsilon_0)/RT})}{\sum_{i=1}^{M}(n_i * e^{-(\varepsilon_i - \varepsilon_0)/RT})} \quad (3)$$

where p is the probability to form the required two Al exchanges sites for Ag_1-Cu_1 hetero SACs, ε_i is the energy of the configuration i, ε_0 is the energy of the most stable configuration of all possible two Al exchange sites, n_i is the number of the configuration i of two Al exchange sites, m is the total number of all the possible configurations of two neighbouring Al exchange sites enabling the formation of Ag_1-Cu_1 hetero SACs, and M is the total number of all the possible configurations of two Al exchanges sites.

Although the total number of the configurations of possible neighbouring two Al exchanges sites is significantly lower than the separated two Al exchange sites, the neighbouring two Al sites are energetically far more stable than the separated two Al exchange sites. In particular, the two Al sites for the formation of the Ag_1-Cu_1 hetero SACs presented in the manuscript is energetically the most favourable configuration. Consequently, the probability of forming the proposed two Al exchanges sites for Ag_1-Cu_1 hetero SACs could arrive at 48.59%, which is the greatest one among all the two Al exchange sites. Hence, it is reasonable to model the two Al sites for the formation of Ag_1-Cu_1 sites.

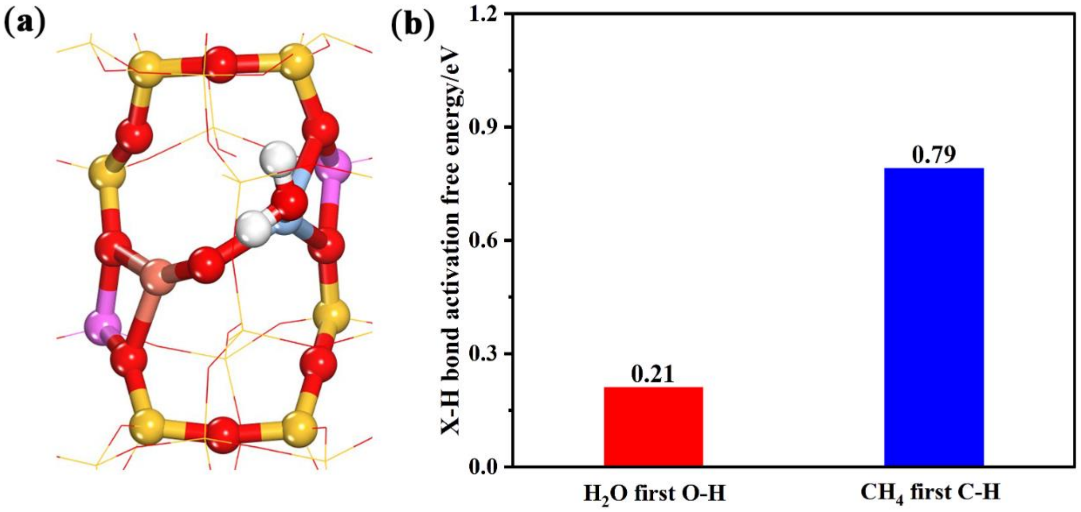

Figure S19. Z[Cu(μ-O)Ag(H_2O)]$^{2+}$ (IM3) site (a) and the activation free energies of the first C-H bond breaking of methane and the first O-H bond breaking of water at Z[Cu(μ-O)Ag(H_2O)]$^{2+}$ (IM3) site (b).

We have calculated the reaction energy and barrier of CH_4 activation by μ-oxo in IM3 (**Figure 4**). It does always be expected that the most destabilized species (μ-oxo in Z[Cu(μ-O)Ag(H_2O)]$^{2+}$ (IM3)) is likely to activate the C-H bond. However, in the presence of H_2O, the μ-oxo in IM3 would prefer to activate H_2O (G_a = 0.21 eV) rather than CH_4 (G_a = 0.79 eV). Moreover, compared with the desorption of H_2O requiring 0.86 eV, the H_2O at Ag site is always preferentially to be activated at IM3. Hence, the presence of H_2O inhibits the μ-oxo at IM3 to activate CH_4.

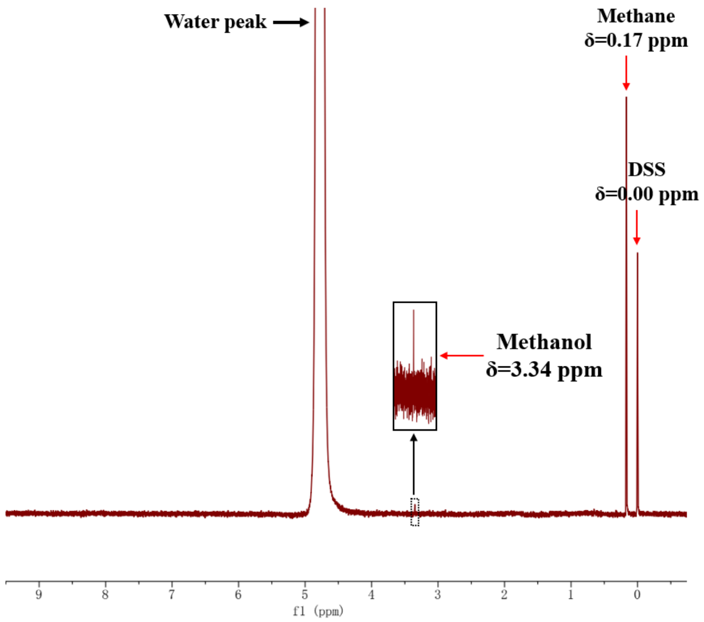

Figure S20. ^{1}**H NMR spectrums of liquid mixtures over** Ag_1**-**Cu_1**/ZSM-5 hetero-SAC after DOM. Reaction conditions: 22 mg catalysts dispersed in 20 ml** H_2O **solution, 3.0 MPa** CH_4 **for 30 min at 70** ^oC**.**

In order to verify the role of H_2O_2 in regenerating the $Z[Cu(OH)]^+[Ag(OH)]^+$ sites, only H_2O is added to the DOM reaction system to detect the liquid products. Under the experimental conditions without H_2O_2 species, only trace methanol can be detected in the liquid phase over Ag_1-Cu_1/ZSM-5 hetero-SAC after DOM reaction, which is in line with the DFT calculation of DOM process that H_2O_2 species can regenerate the $Z[Cu(OH)]^+[Ag(OH)]^+$ sites and promote the DOM process.

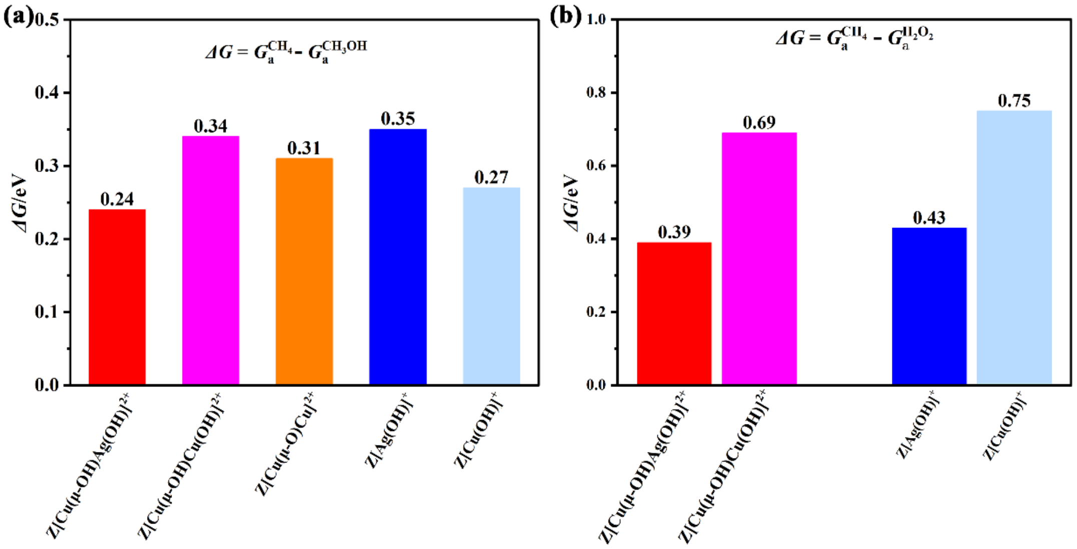

Figure S21. The difference in the activation free energy between the C-H bond of CH_4 and the C-H bond of CH_3OH at Z[Cu(μ-OH)Ag(OH)]$^{2+}$, Z[Cu(μ-OH)Cu(OH)]$^{2+}$, Z[Cu(μ-O)Cu]$^{2+}$, Z[Ag(OH)]$^{+}$, and Z[Cu(OH)]$^{+}$ sites (a); The difference in the activation free energy between the C-H bond of CH_4 and the C-H bond of H_2O_2 at Z[Cu(μ-OH)Ag(OH)]$^{2+}$, Z[Cu(μ-OH)Cu(OH)]$^{2+}$, Z[Ag(OH)]$^{+}$, and Z[Cu(OH)]$^{+}$ sites (b). $G_a^{CH_4}$ and $G_a^{CH_3OH}$ respectively denote the free energy barriers of the first C-H bond breaking of methane and methanol.

For the locally optimized structures of active sites in ZSM-5 (Z[Cu(OH)Ag(OH)]$^{2+}$, Z[Cu(μ-OH)Cu(OH)]$^{2+}$, Z[Cu(μ-O)Cu]$^{2+}$ sites and mononuclear Z[Cu(OH)]$^{+}$ and Z[Ag(OH)]$^{+}$ sites as shown in **Figure 5a** in the main manuscript, we calculated and compared the difference in the activation free energies of the C-H bond of CH_4 and CH_3OH, and the O-H bond of H_2O_2. Previous study[8] has shown that their difference ($\Delta G = G_a^{CH_4} - G_a^{CH_3OH}$) is related to the selectivity between direct methane to methanol and the deep oxidation of methanol. The less positive the difference, the lower probability for the deep oxidation of produced methanol at the same conversion of methane. Therefore, their difference was used herein as an index to evaluate the selectivity of methane to methanol. The minimum C-H bond activation energy barrier gap between CH_4 and CH_3OH at Z[Cu(μ-OH)Ag(OH)]$^{2+}$ indicates that the synergistic effects between Cu and Ag dual single atoms could also improve the selectivity towards the formation of methanol compared with Ag_1/ZSM-5 SAC or Cu_1/ZSM-5 SAC. The difference between the C-H bond activation energy barrier of CH_4 and the C-H bond activation energy barrier of CH_3OH proves that the introduction of silver species can improve the selectivity of Cu-based zeolite catalyst and is more conducive to the formation of methanol, which is in line with the catalytic performances of DOM (**Figure 3a** in the main manuscript). In addition, the difference between the C-H bond activation energy barrier of CH_4 and the O-H bond activation energy barrier of H_2O_2 shows that the copper and silver synergistic effect narrows the energy barrier gap, thus improving the competitiveness of CH_4 to a certain extent in the competitive reaction process of the active site.

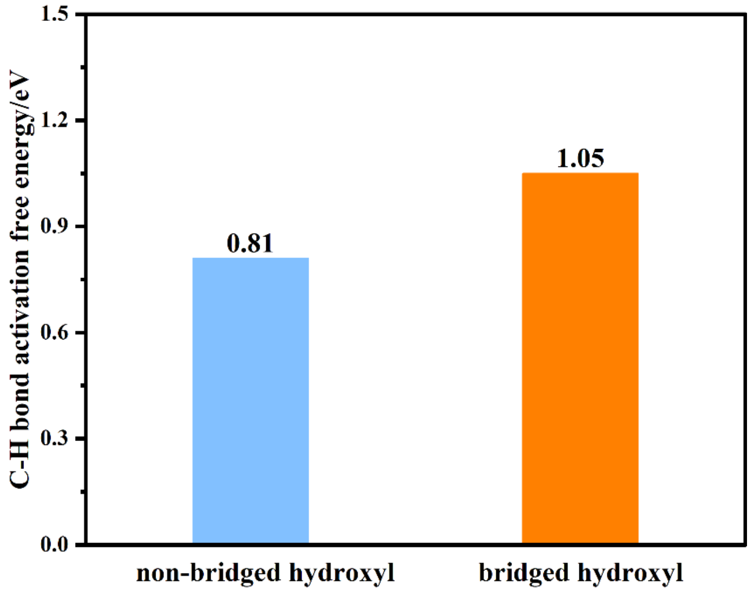

Figure S22. The activation free energies of the first C-H bond breaking of methane by the non-bridge hydroxyl and bridge hydroxyl of Z[Cu(μ-OH)Ag(OH)]$^{2+}$.

As shown in **Table S4**, the calculated Bader charge of μ-hydroxyl oxygen at the Z[Cu(μ-OH)Ag(OH)]$^{2+}$ site is -1.08 |e|, which is close to -1.12 |e| of the O^{2-} in H_2O, indicating the formation of OH^{-} without the radical-like character. The further C-H bond activation calculation results further demonstrate the lower activity of μ-hydroxyl compared with the non-bridged hydroxyl with the radical-like character. The activation of C-H bond of methane by the bridged hydroxyl at the Z[Cu(μ-OH)Ag(OH)]$^{2+}$ site needs to overcome the free energy barrier of 1.05 eV, which is 0.24 eV higher than that by non-bridged hydroxyl at Ag site. In sum, the non-bridged hydroxyl with the radical character rather than the bridged hydroxyl is the reactive oxygen species for the C-H bond activation over Ag_1-Cu_1/ZSM-5.

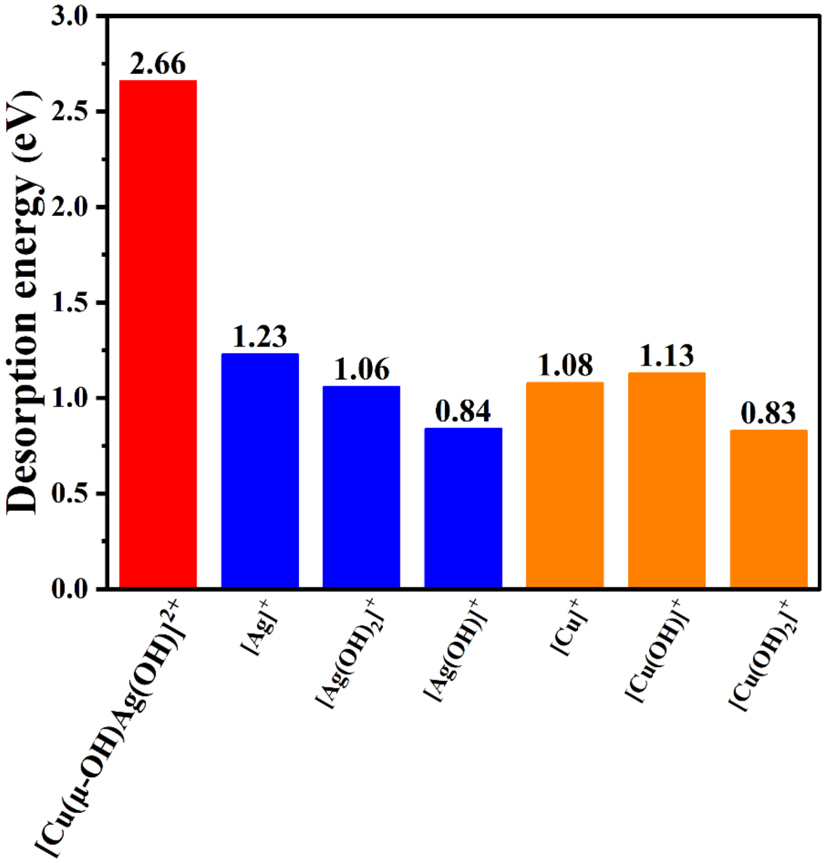

Figure S23. The desorption energies of active sites [Cu(μ-OH)Ag(OH)]$^{2+}$, [Ag]$^{+}$, [Ag(OH)$_2$]$^{+}$, [Ag(OH)]$^{+}$, [Cu]$^{+}$, [Cu(OH)$_2$]$^{+}$, and [Cu(OH)]$^{+}$anchored over the ZSM-5 zeolite framework.

The DFT calculations also show that the desorption of Ag or Cu species away from the zeolite skeleton, evidently lower than that of Ag_1-Cu_1 dual sites (2.66 eV), which indicates that the sole Ag-based and Cu-based active sites are less stable. Those DFT results corroborate the experimental results that the Ag and Cu are prone to aggregating in the reaction process of DOM over Ag_1/ZSM-5 SAC and Cu_1/ZSM-5 SAC.

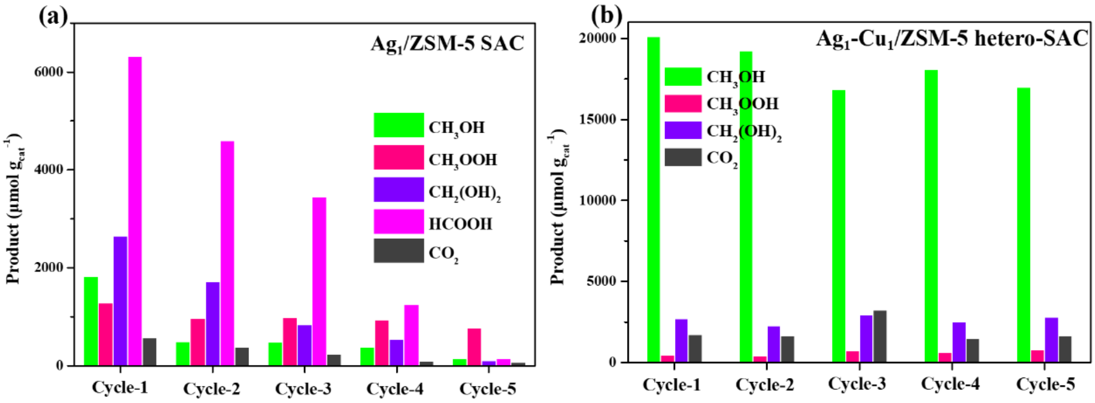

Figure S24. Reaction cycles of DOM coupling on Ag_1/ZSM-5 SAC (a) and Ag_1-Cu_1/ZSM-5 hetero-SAC (b). Reaction conditions: 22mg catalysts dispersed in 21.05 ml 0.489 M H_2O_2 solution, 3.0 MPa CH_4 for 30 min at 70 °C.

The stability experiments suggest that the productivity of C1 oxygenates from Ag_1/ZSM-5 SAC drops dramatically by 90.3% after 5 cycles while the productivity of C1 oxygenates over Ag_1-Cu_1/ZSM-5 hetero-SAC only drops by 11.1% after 5 cycles, which confirms that the stability of Ag_1-Cu_1/ZSM-5 hetero-SAC is better than that of Ag_1/ZSM-5 SAC, corroborating the conclusion of DFT simulation.

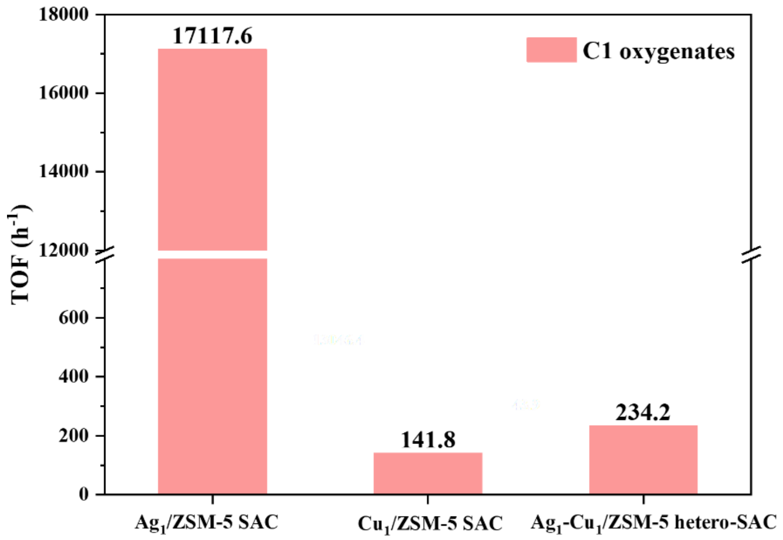

Figure S25. Intrinsic activity of Ag_1/ZSM-5 SAC, Cu_1/ZSM-5 SAC and Ag_1-Cu_1/ZSM-5 hetero-SAC. Reaction conditions: 22 mg catalysts dispersed in 21.05 ml 0.489 M H_2O_2 solution, 3.0 MPa CH_4 for 30 min at 70 ^oC.

As clearly shown in the intrinsic activity, sole silver single atoms loaded on ZSM-5 can significantly improve the activity of DOM, which is in line with the conclusion from DFT calculation in **Figure 5b** (the first C-H bond activation of CH_4 on Ag single core is much lower than the others) in the main manuscript. Those results further confirm that single Ag atoms supported on ZSM-5 have much better performance of DOM.

Turnover frequency (TOF) is defined as:

$$TOF\ (h^{-1}) = \frac{\text{C1 Products (mol)}}{\text{Metal (mol)} \bullet \text{Time (h)}} \quad (4)$$

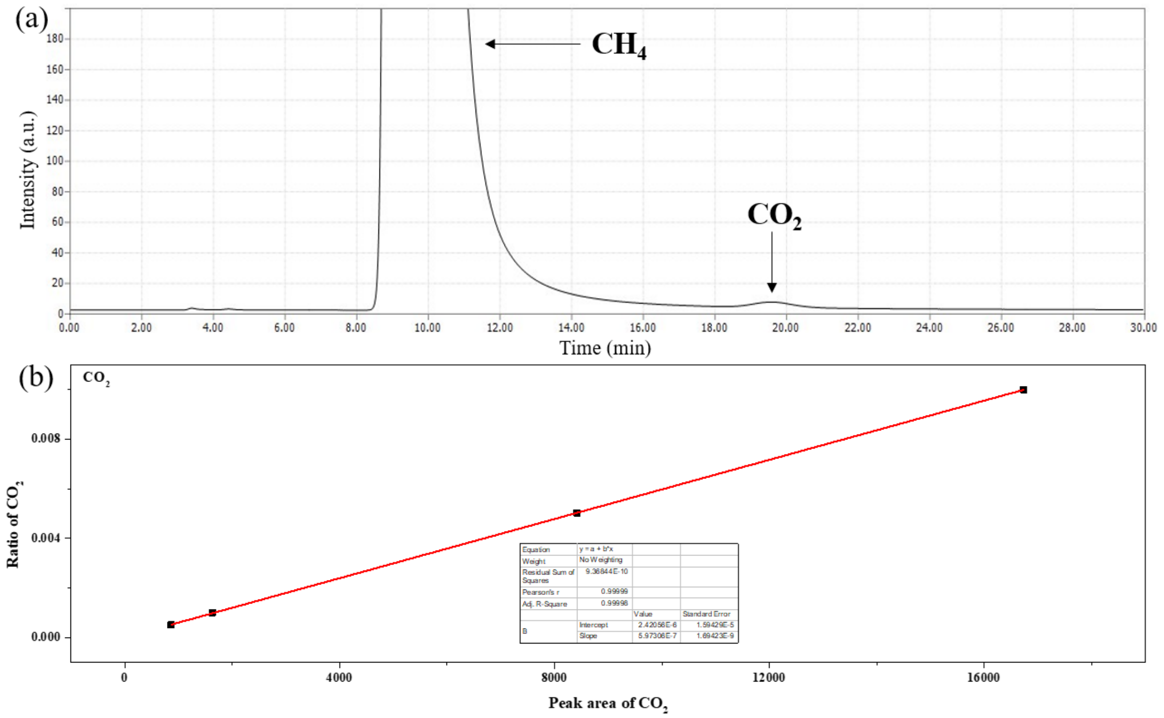

Figure S26. (a) A typical chromatogram of Ag_1-Cu_1/ZSM-5 hetero-SAC after DOM. (b) Standard curve for CO_2 quantification. The ordinate (Y) represents the content of CO_2 and the abscissa (X) represents the peak area of CO_2 in the chromatogram.

Only CH_4 (8.9 min) and CO_2 (19.5 min) could be detected in GC of gas mixture after DOM (**Figure S26a**). In order to calculate the output of CO_2 after DOM reaction, we established the standard curve by customizing the electronic grade high-purity standard CO_2 gas. As shown in **Figure S26b**, the R-square of the curve was 0.99998, which indicates the degree of fitting was reliable. The amount of CO_2 is calculated from the peak area in the chromatogram and the standard curve.

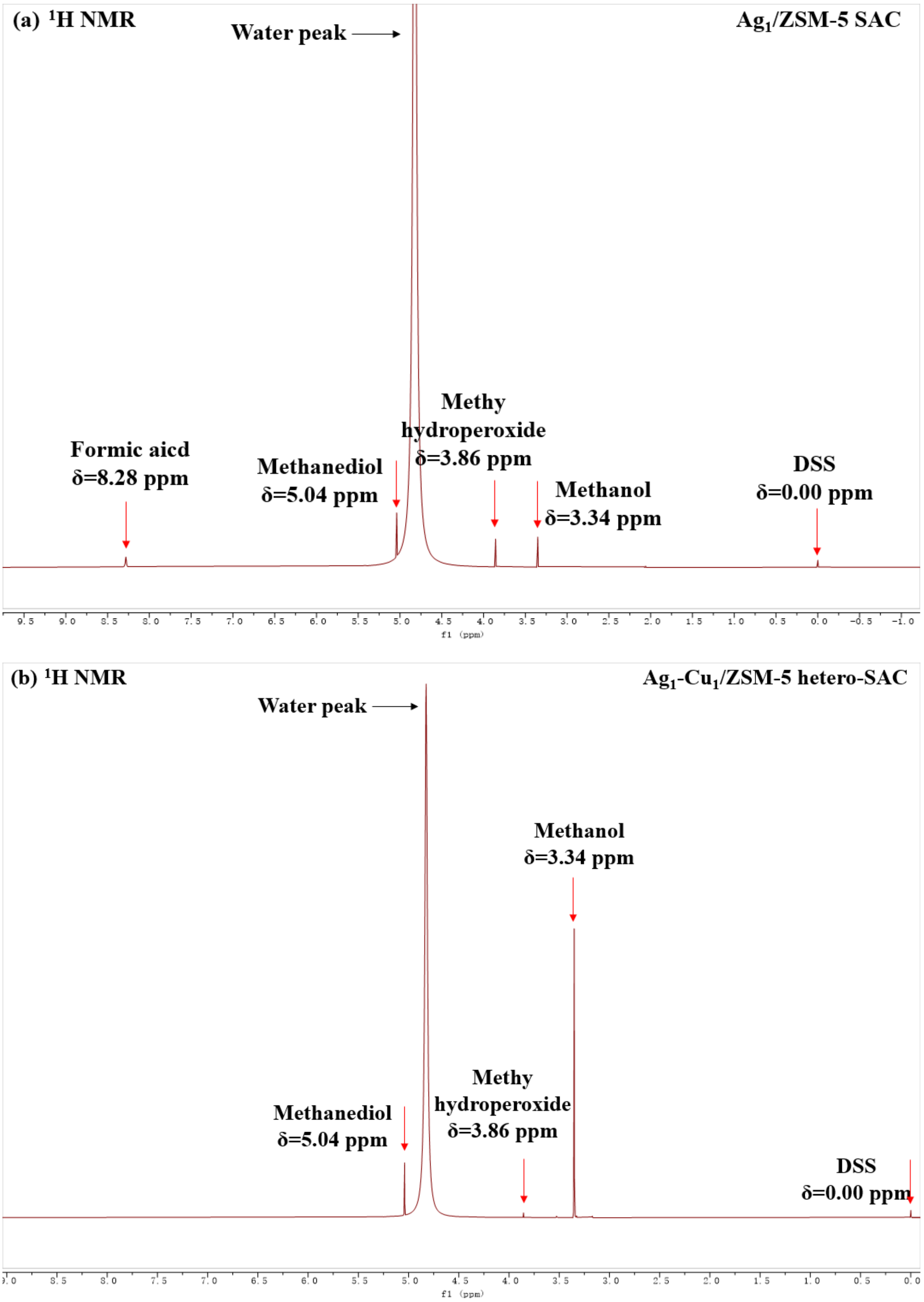

Figure S27. Typical ¹H NMR spectrums of liquid mixtures over (a) Ag_1/ZSM-5 SAC and (b) Ag_1-Cu_1/ZSM-5 hetero-SAC after DOM. Reaction conditions: 22 mg catalysts dispersed in 21.05 ml 0.489 M H_2O_2 solution, 3.0 MPa CH_4 for 30 min at 70 ºC.

All ¹H NMR spectra were calibrated by phase correction and baseline before quantitative calculation of products for the DOM.

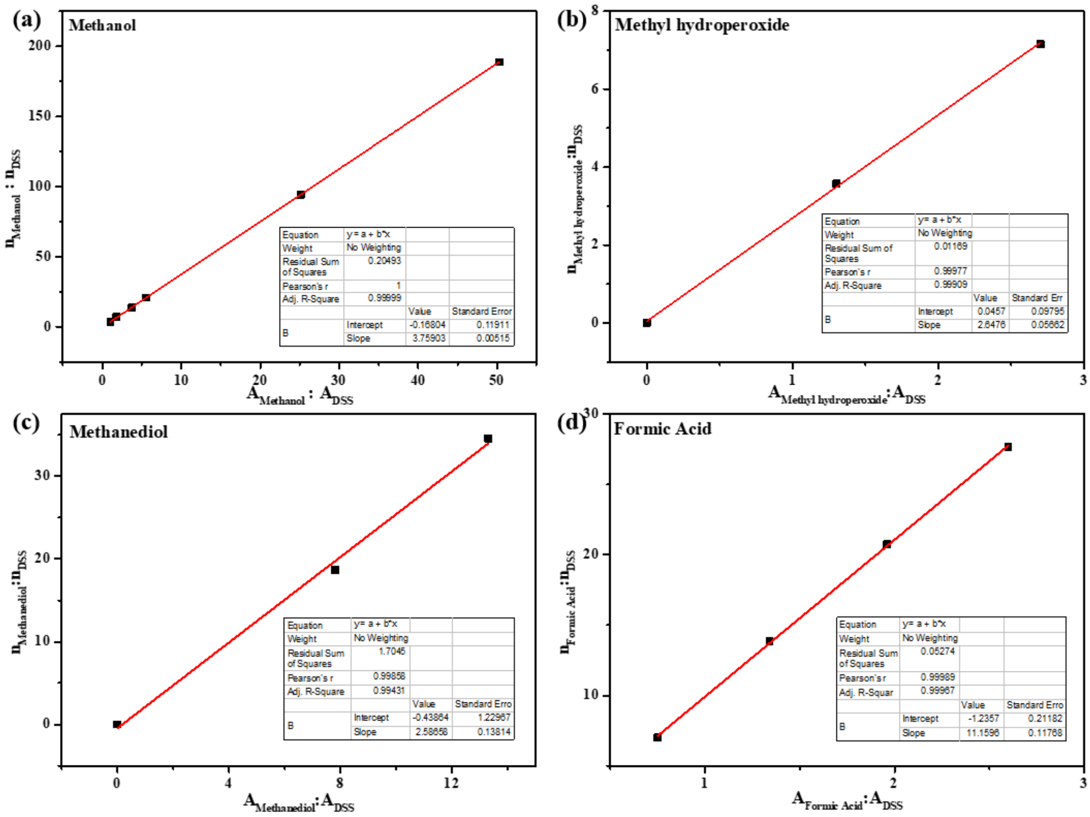

Figure S28. The standard curves of (a) methanol, (b) methyl hydroperoxide, (c) methanediol, (d) formic acid for calculating liquid product after DOM.

3-(trimethylsilyl)-1-propanesulfonic acid sodium salt (DSS) was used as a calibration standard to build standard curves. The X-axis represents the peak area ratio of the product vs DSS ($A_{product}:A_{DSS}$) in the ^{1}H NMR spectrum. The Y-axis represents the amount of the product vs DSS ($n_{product}:n_{DSS}$). The R-square of standard curves are all higher than 0.99, indicating the degree of fitting is reliable.

Table S1. Catalytic performance of the most promising catalysts in the DOM reported in literature recently.

Catalyst	Loading	P	t	T	Yield of CH_3OH	S^a of CH_3OH	Oxidant/Phase	Notes
	(wt.%)	(bar)	(h)	(°C)	(μmol•g_{cat}^{-1})	(%)		
**Ag_1-Cu_1/ZSM-5 hetero-SAC**	**0.0047(Ag)***	**30**	**0.5**	**70**	**20115**	**81**	**H_2O_2/L.[b]**	**This work**
Cu-Fe(2/0.1)/ZSM-5	0.13(Fe)*	30	0.5	50	3891	80	H_2O_2/L.	Ref [2]
Cu_1/ZSM-5	0.34*	30	0.5	50	3139	74	H_2O_2/L.	Ref [5]
Cu-Fe/ZSM-5	2.5 (Fe)	30	0.5	50	4100	85	H_2O_2/L.	Ref [9]
Fe/ZSM-5	2.5	30	0.5	50	593	12	H_2O_2/L.	Ref [9]
Au-Pd/TiO_2	1 (1:1)	30	0.5	50	35	12	H_2O_2/L.	Ref [10]
Pd_1/ZSM-5	0.01	30	0.5	50	277	7	H_2O_2/L.	Ref [11]
Rh_1/ZrO_2	0.3	30	0.5	70	312	64	H_2O_2/L.	Ref [12]
Au-Pd colloid	100	30	0.5	50	3000	14	H_2O_2+O_2/L.	Ref [13]
FeN_4/GN	2.7*	20	10	25	128	5	H_2O_2/L.	Ref [14]
Fe/ZSM-5 (66)	0.03*	30	0.5	80	325	1.2	H_2O_2/L.	Ref [15]
Cr_1/TiO_2	1	30	1	50	377	8	H_2O_2/L.	Ref [16]
Rh_1/CeO_2 NWs	0.29*	5	1	50	2879	75	H_2O_2/L.	Ref [17]
FeO_x/TiO_2	0.33*	-	3	25	1050	90	H_2O_2/L.	Ref [18]
Fe-HZ5-TF	0.4	30	0.42	50	1267	5	H_2O_2/L.	Ref [19]
Fe-HZ5-C	0.25	30	0.42	50	641	8	H_2O_2/L.	Ref [19]
W-SA-PCN-7.5	0.47*	5	5	25	1050	21	H_2O_2/L.	Ref [20]
UiO-66-H	-	30	0.5	50	20	5.4	H_2O_2/L.	Ref [21]

a: Selectivity b: Liquid.

*: The actual loading of metal determined by ICP.

Table S2. EXAFS fitting results for Ag_1-Cu_1/ZSM-5 hetero-SAC according to the standard crystal model provided by DFT.

Sample	Path	CN	R_{fit} (Å)	R_{dft} (Å)	σ^2 (Å^2)	R-factor	ΔE (eV)
Ag_1-Cu_1/ZSM-5 hetero-SAC	Cu-O_{OH}	1	1.91	1.91	0.0011	0.015	-4.8
	Cu-O_{FM}	2	1.98	1.98/1.97	0.0031		

CN is the coordination number; R_{fit} is fitting distance; R_{real} is real distance in the model provided by DFT calculation; σ^2 is Debye-Waller factor.

Table S3. The DFT calculation results of Bader charge analysis for Cu species over Ag_1-Cu_1/ZSM-5 hetero-SAC and CuO.

Sample	Bader charge (e)
Ag_1-Cu_1/ZSM-5 hetero-SAC	0.98
CuO	1.08
Cu_2O	0.54

Table S4. Bader charge analysis of oxygen at different sites.

site	Position of O	Bader charge (|e|)	Valence state
$Z[AgOH]^{+}[CuOH]^{+}$	OH (Ag)	-0.74	HO·
	OH (Cu)	-1.01	HO^{-}
$Z[Cu(\mu\text{-}OH)Ag(OH)]^{2+}$	OH (Ag)	-0.79	HO·
	μ-OH	-1.08	HO^{-}
$Z[Pd(\mu\text{-}OH)Cu(OH)]^{2+}$	OH (Cu)	-0.99	HO^{-}
	μ-OH	-1.01	HO^{-}
$Z[Cu(OH)]^{+}$	OH (Cu)	-1.08	HO^{-}
H_2O_2	-	0.62	O^{-}
H_2O	-	1.12	O^{2-}

According to the recent work[22], the formal charge of the oxygen species could be identified based on the reference Bader charges of the O^{2-} in H_2O (-0.62 |e|) and the O^{-} in H_2O_2 (-1.12 |e|). The Bader charge of the initial site $Z[AgOH]^{+}[CuOH]^{+}$ and the active site $Z[Cu(\mu\text{-}OH)Ag(OH)]^{2+}$ was calculated, and the charge of the key oxygen species was listed from the above table, further indicating the state of the adsorbed hydroxyl group.

Table S5. Nominal and actual M & Cu loading of different catalysts.

Sample	Nominal content (M, wt. %)	Actual content (M, wt. %)	Nominal content (Cu, wt. %)	Actual content (Cu, wt. %)
Pd_1-Cu_1/ZSM-5	0.01	0.0051	2	1.22
Ir_1-Cu_1/ZSM-5	0.01	0.0077	2	1.29
Pt_1-Cu_1/ZSM-5	0.01	0.0059	2	1.39
Rh_1-Cu_1/ZSM-5	0.01	0.0034	2	1.46
Au_1-Cu_1/ZSM-5	0.01	0.0087	2	1.37
Ag_1-Cu_1/ZSM-5	0.01	0.0047	2	1.26

***:** The actual loading of metal determined by ICP-OES.

Table S6. Intensity of hydroxyl radicals signal in EPR spectra.

Catalyst	Intensity of hydroxyl radical signal (a. u.)*				Total Intensity (a. u.)	Normalization (a. u.)
	a	b	c	d		
Ag_p-Cu_p/ZSM-5	285	585	552	267	1689	~1.6
Ag_1-Cu_p/ZSM-5	846	1831	1834	840	5341	~5.2
Ag_1 /ZSM-5 SAC	547	1153	1112	539	3351	~3.2
Ag_1-Cu_1/ZSM-5 hetero-SAC	170	360	345	175	1045	1

*: The signal intensity of hydroxyl radical is determined by the area of the integral peak.

The normalized data is based on the hydroxyl radical activity of Ag_1-Cu_1/ZSM-5 hetero-SAC is defined as "1". The hydroxyl radical activity produced by dissociating hydrogen peroxide of Ag_p-Cu_p/ZSM-5, Ag_1-Cu_p/ZSM-5 and Ag_1/ZSM-5 SAC samples are 1.6, 5.2 and 3.2 times higher than that of Ag_1-Cu_1/ZSM-5 hetero-SAC samples.

Table S7. The standard free energy of activation and the free energy change of each key elementary step in the micropores of ZSM-5 solution at 343 K.

Elementary steps	$\Delta G^{\neq}$ (eV)	ΔG (eV)
$CH_4 + \cdot OH \rightarrow \cdot CH_3 + H_2O$	0.96	-0.68
$CH_3OH + \cdot OH \rightarrow \cdot CH_2OH + H_2O$	0.57	-1.03
$CH_3OH + OH\cdot \rightarrow CH_3O\cdot + H_2O$	0.71	-0.67
$CH_2OH\cdot + H_2O_2 \rightarrow CH_4O_2 + OH\cdot$	0.45	-2.19

Table S8. Actual Ag loading of used Ag_1/ZSM-5 SAC.

Sample	Ag content* (wt.%)
Ag_1/ZSM-5 SAC Fresh	0.0053
Ag_1/ZSM-5 SAC After cycle 1	0.0039
Ag_1/ZSM-5 SAC After cycle 5	0.0002

***:** The actual loading of metal determined by ICP-OES.

Table S9. Actual Ag & Cu loading of used Ag_1-Cu_1/ZSM-5 hetero-SAC.

Sample	Ag content* (wt.%)	Cu content* (wt.%)
Ag_1-Cu_1/ZSM-5 hetero-SAC Fresh	0.0047	1.26
Ag_1-Cu_1/ZSM-5 hetero-SAC After cycle 1	0.0046	1.25
Ag_1-Cu_1/ZSM-5 hetero-SAC After cycle 5	0.0046	1.23

***:** The actual loading of metal determined by ICP-OES.

Table S10. Actual Ag & Cu loading of different dispersion catalysts.

Sample	Ag content* (wt.%)	Cu content* (wt.%)
Ag_p-Cu_p/ZSM-5	0.0052	1.29
Ag_1-Cu_p/ZSM-5	0.0043	1.11
Ag_1-Cu_1/ZSM-5 hetero-SAC	0.0047	1.26

***:** The actual loading of metal determined by ICP-OES.

References

1. Negri, C.; Signorile, M.; Porcaro, N. G.; Borfecchia, E.; Berlier, G.; Janssens, T. V. W.; Bordiga, S., *Appl. Catal. A:Gen.* **2019**, *578*, 1.

2. Yu, T.; Li, Z.; Lin, L.; Chu, S.; Su, Y.; Song, W.; Wang, A.; Weckhuysen, B. M.; Luo, W., *ACS Catal.* **2021**, *11*, 6684.

3. Li, Y.; Deng, J.; Song, W.; Liu, J.; Zhao, Z.; Gao, M.; Wei, Y.; Zhao, L., *J. Phys. Chem. C* **2016**, *120*, 14669.

4. Centi, G.; Fazzini, F.; Galli, A., *Res. Chem. Intermed.* **1998**, *24*, 541.

5. Tang, X.; Wang, L.; Yang, B.; Fei, C.; Yao, T.; Liu, W.; Lou, Y.; Dai, Q.; Cai, Y.; Cao, X. M.; Zhan, W.; Guo, Y.; Gong, X. Q.; Guo, Y., *Appl. Catal. B: Environ.* **2021**, *285*, 119827.

6. Punnoose, A.; Seehra, M. S.; Dunn, B. C.; Eyring, E. M., *Energy Fuels* **2002**, *16*, 182.

7. Qu, W.; Yuan, H.; Ren, Z.; Qi, J.; Xu, D.; Chen, J.; Chen, L.; Yang, H.; Ma, Z.; Liu, X.; Wang, H.; Tang, X., *Angew. Chem. Int. Ed.* **2022**, *61*, e202212703.

8. Latimer, A. A.; Kakekhani, A.; Kulkarni, A. R.; Nørskov, J. K., *ACS Catal.* **2018**, *8*, 6894.

9. Hammond, C.; Forde, M. M.; Ab Rahim, M. H.; Thetford, A.; He, Q.; Jenkins, R. L.; Dimitratos, N.; Lopez-Sanchez, J. A.; Dummer, N. F.; Murphy, D. M.; Carley, A. F.; Taylor, S. H.; Willock, D. J.; Stangland, E. E.; Kang, J.; Hagen, H.; Kiely, C. J.; Hutchings, G. J., *Angew. Chem. Int. Ed.* **2012**, *51*, 5129.

10. Ab Rahim, M. H.; Forde, M. M.; Jenkins, R. L.; Hammond, C.; He, Q.; Dimitratos, N.; Lopez-Sanchez, J. A.; Carley, A. F.; Taylor, S. H.; Willock, D. J.; Murphy, D. M.; Kiely, C. J.; Hutchings, G. J., *Angew. Chem. Int. Ed.* **2013**, *52*, 1280.

11. Huang, W.; Zhang, S.; Tang, Y.; Li, Y.; Nguyen, L.; Li, Y.; Shan, J.; Xiao, D.; Gagne, R.; Frenkel, A. I.; Tao, F. F., *Angew. Chem. Int. Ed.* **2016**, *55*, 13441.

12. Kwon, Y.; Kim, T. Y.; Kwon, G.; Yi, J.; Lee, H., *J. Am. Chem. Soc.* **2017**, *139*, 17694.

13. Agarwal, N.; Freakley, S. J.; McVicker, R. U.; Althahban, S. M.; Dimitratos, N.; He, Q.; Morgan, D. J.; Jenkins, R. L.; Willock, D. J.; Taylor, S. H.; Kiely, C. J.; Hutchings, G. J., *Science* **2017**, *358*, 223.

14. Cui, X.; Li, H.; Wang, Y.; Hu, Y.; Hua, L.; Li, H.; Han, X.; Liu, Q.; Yang, F.; He, L.; Chen, X.; Li, Q.; Xiao, J.; Deng, D.; Bao, X., *Chem* **2018**, *4*, 1902.

15. Zhu, K.; Liang, S.; Cui, X.; Huang, R.; Wan, N.; Hua, L.; Li, H.; Chen, H.; Zhao, Z.; Hou, G.; Li, M.; Jiang, Q.; Yu, L.; Deng, D., *Nano Energy* **2021**, *82*,105718.

16. Shen, Q.; Cao, C.; Huang, R.; Zhu, L.; Zhou, X.; Zhang, Q.; Gu, L.; Song, W., *Angew. Chem. Int. Ed.* **2020**, *59*, 1216.

17. Bai, S.; Liu, F.; Huang, B.; Li, F.; Lin, H.; Wu, T.; Sun, M.; Wu, J.; Shao, Q.; Xu, Y.; Huang, X., *Nat. Commun.* **2020**, *11*, 954.

18. Xie, J.; Jin, R.; Li, A.; Bi, Y.; Ruan, Q.; Deng, Y.; Zhang, Y.; Yao, S.; Sankar, G.; Ma, D.; Tang, J., *Nat. Catal.* **2018**, *1*, 889-896.

19. Cheng, Q.; Li, G.; Yao, X.; Zheng, L.; Wang, J.; Emwas, A.-H.; Castaño, P.; Ruiz-Martínez, J.; Han, Y., *J. Am. Chem. Soc.* **2023,** *145*, 5888.

20. Wang, Y.; Zhang, J.; Shi, W.-X.; Zhuang, G.-L.; Zhao, Q.-P.; Ren, J.; Zhang, P.; Yin, H.-Q.; Lu, T.-B.; Zhang, Z.-M., *Adv. Mater.* **2022,** *34*, 2204448.

21. Fang, G.; Hu, J.-N.; Tian, L.-C.; Liang, J.-X.; Lin, J.; Li, L.; Zhu, C.; Wang, X., *Angew. Chem. Int. Ed.* **2022,** *61*, e202205077.

22. Rosen A S, Notestein J M, Snurr R Q., *Angew. Chem. Int. Ed.* **2020**, *59*, 19494.
